# Retaliatory killing and human perceptions of Madagascar’s largest carnivore and livestock predator, the fosa (*Cryptoprocta ferox*)

**DOI:** 10.1371/journal.pone.0213341

**Published:** 2019-03-15

**Authors:** Samuel D. Merson, Luke J. Dollar, Paul J. Johnson, David W. Macdonald

**Affiliations:** 1 Zoological Society of London, Outer Circle, Regent’s Park, London, United Kingdom; 2 Nicholas School of the Environment, Duke University, Durham, North Carolina, United States of America; 3 Department of Environment & Sustainability, Center for the Environment, Catawba College, Salisbury, North Carolina, United States of America; 4 Wildlife Conservation Research Unit, Department of Zoology, University of Oxford, Tubney, Oxfordshire, United Kingdom; University of Minnesota, UNITED STATES

## Abstract

Fosas (*Cryptoprocta ferox*) are Madagascar’s largest carnivores, occupying much of the island’s forested landscape. This study provides the first evaluation of fosas’ conflict with humans, a problem for many small and medium sized carnivores worldwide. We examined fosas’ predation of poultry, and the subsequent retaliatory killing. Over 1750 households were interviewed across four regions, encompassing Madagascar’s major forest types (deciduous/rainforest) and protected area classifications (national park, reserve and unprotected forest). Predation by fosa was the third highest reported cause (15%) of poultry mortality, with little evidence that coops were effective in reducing predation. Predation of poultry was more prevalent in deciduous forests, and most common during the evenings of the dry season. Over half of all interviewees said they disliked fosas, with loss of poultry the most commonly stated reason. Respondents’ that had suffered poultry depredation and those with lower educational attainment were more likely to dislike fosas. Interviewees that disliked fosas and those that were wealthier were most likely to report having killed a fosa. A minimum of thirty fosas was killed in retaliation by our respondents during the year before the interviews. Given that the fosa population is in decline, and most of Madagascar’s forests are likely to be too small to support sustainable populations, these killings may be detrimental to vulnerable sub-populations. These results shed insight into the cultural perceptions and predation patterns of a medium sized carnivore, with relevance to worldwide human-wildlife conflict of often overlooked smaller carnivores. We suggest that educational programs, guard dogs, poultry disease vaccinations and robust coop construction may be effective for improving attitudes and reducing retaliatory killing.

## Introduction

Carnivore populations are in global decline, with human conflict and retaliatory killing a leading cause [1–4]. While larger carnivores may be particularly at risk, owing to larger home ranges, low population densities, and low fecundity [4, 5], conflict with carnivore populations is not limited to larger species. Across the globe many small to medium carnivores are also persecuted, most often for their predation upon domestic poultry [6, 7].

Madagascar’s carnivores, the Eupleridae, have been described as one of the most understudied and threatened group of carnivores worldwide [8]. The Eupleridae’s largest carnivore, the fosa (*Cryptoprocta ferox*) weighs on average 6–7 kg [9, 10] and are found across Madagascar’s forested landscape. They are thought to be a keystone species due to their predation of lemurs and other species [11, 12]. Fosas occupy home ranges of up to 50 km^2^ [13] at population densities of 0.17–0.22 individual/km^2^ in rainforests and deciduous forests [14, 15]. It is this extensive ranging and predation on larger prey that leads them to encounter humans and their poultry. Knowledge of this relationship however, has hitherto been largely anecdotal.

Fosas are classified as a Category 1 Class 2 protected species under Madagascar’s National Classification for Animal Species. Unlike a Category 1 Class 1 species that are strictly protected, fosas may however be hunted under authorisation, commercial, sport or regulatory conditions. These legislative discrepancies are however unlikely to be known my most Malagasy [16, 17].

Fosas have reportedly been killed for straying into villages where they may be perceived to be a threat to poultry [9, 18, 19] and other small livestock such as lambs [20]. Recent research in villages surrounding Ranomafana National Park [21] suggests fosas are not believed to affect many poultry keepers living at distance from the forest, with less than 1% of poultry predation attributed to fosas. However, individual attacks can be serious, with local farmers reporting incidents where fosas have killed all of their poultry once gaining access to their coop [18]. So called ‘surplus killing’, is likely to promote negative attitudes towards the predator, as has been observed of the red fox in the UK [22].

Negative attitudes to fosas are not only attributable to poultry predation, but also to taboos (known as *fady*). The most commonly cited is the belief that they consume the remains of villagers’ ancestor’s [19, 23]. Their resemblance to dogs is also a negative influence [20], perhaps underpinned by a general dislike of dogs, as recorded in Ranomafana National Park [24]. There are currently no published studies verifying these anecdotal accounts of human persecution of fosas. Given fosas’ life history characteristics, potential persecution raises questions as to their long-term persistence in a landscape shared with humans.

This study examines fosas’ impact across four regions, encompassing a representative sample of Madagascar’s protected and unprotected areas. Our objectives were to: 1) Identify respondents’ livestock ownership practices; 2) Examine the frequency and cost of fosa predation relative to other causes of poultry mortality; 3) Identify fosas’ primary temporal and seasonal periods of poultry predation; 4) Estimate the annual numbers of fosas killed and examine variation among the four regions; 5) Identify the circumstances where predation of domestic poultry and therefore retaliatory killing of fosas is most likely, and 6) explore interviewee attitudes towards fosas. This information is fundamental for evaluating the retaliatory killing of fosas, and medium size predators in general, and understanding its socio-economic and cultural drivers.

## Materials and methods

### Study area

**Fig 1:**
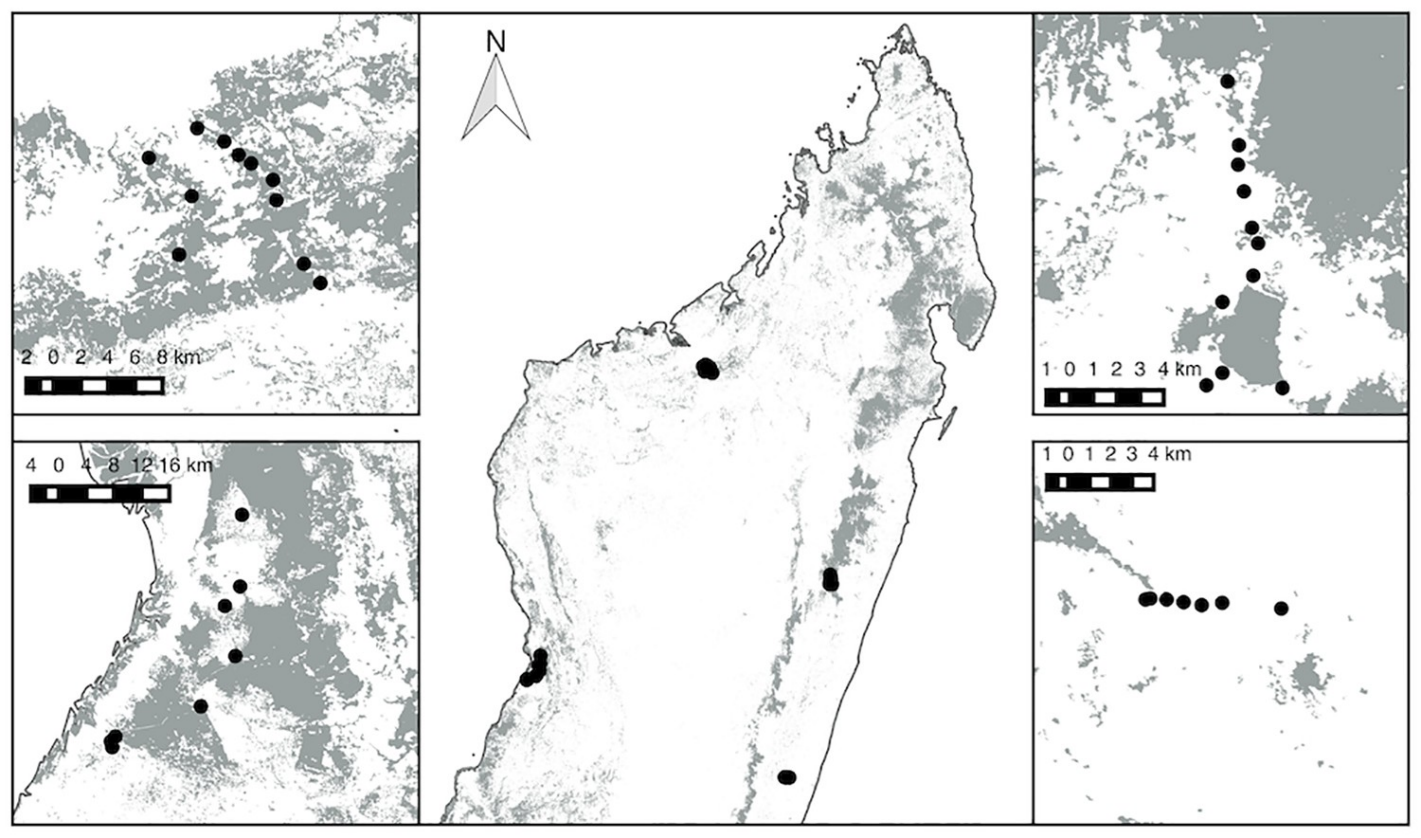
Map of the four surveys regions. Clockwise from top-left, and in order are Boeny, Moramanga, Vatovavy-Fitovinany and Menabe (villages denoted in inserts by black circles).

Surveys were conducted across four sites in Madagascar during April–July 2014, and May—August 2015. Sites were chosen to encompass a representative sample of two of the fosas’ most extensive habitat types (deciduous and rainforest) (Fig 1). Madagascar has a hierarchy of protected areas ranging from; Category I, Strict Natural Reserve, forbidding visitor entry; Category II, National Park, managed to allow public entry; and Category III, Special Reserve which are preserved purely for conservation, with a minimal tourist focus [25]. Our four sites incorporated two National Parks, one Special Reserve, one Private Reserve, and two unprotected areas (Table 1).

**Table 1:** Summary of our study regions, and the results of our three models. Including: percentage of households (HH) that experienced fosa poultry predation; percentage of households that killed a fosa; and the average household attitude score.

Forest Type	Region	Protection Status	Forest Size (km^2^)	Total Villages	Households Interviewed	Fosa Predation (HH%)	Fosas Killed (HH%)	Mean HH Attitude Score
Deciduous	Boeny	National Park (Ankarafantsika)	1,350	8	363	22%	5%	2.83
	Menabe	Special Reserve (Andranomena)	64	2	190	16%	4.11%	3.03
	Menabe	Private Reserve (Kirindy)	710	2	266	41%	1.79%	2.74
	Menabe	Unprotected (Ambadira)	710	1	206	37%	1.94%	2.65
Rainforest	Moramanga	National Park (Andasibe-Mantadia)	155	6	362	11%	5.96%	2.43
	Vatovavy-Fitovinany	Unprotected (various)	Various < 10	5	359	7%	0.42%	2.56

#### Deciduous forest: National Park

Located in the Boeny region, Ankarafantsika National Park (ANP)(16°14’ S latitude, 46°55’ E longitude) is Madagascar’s largest (1350 km^2^) tract of deciduous forest. ANP is surrounded by a population of over 37,000 human inhabitants in 133 villages and hamlets [26]. A road intersects the park’s southwest and is flanked by six villages. These, and two villages to their southwest are the only villages inside the forest.

#### Deciduous forest: Special and private reserve, unprotected area

The largest fragments of deciduous forest are in the central western region of Menabe. Three forests are situated along a south-north axis in Menabe’s north. Andranomena Special Reserve (ASR)(20°17’ S latitude, 44°50’ E longitude) contains 64.20 km^2^ of deciduous forest with two large villages flanking its perimeter. North of Andranomena is the privately managed Kirindy reserve (20°04’ S latitude, 44°66’ E longitude), a former forestry concession and now a centre of research. Lastly Ambadira, unprotected and the most northerly forest, is bordered by one large village. During 2008–2010 it experienced annual deforestation rates as high as 13%, often a result of agricultural conversion [27].

#### Rainforest: National Park

Covering over 155 km^2^, Andasibe-Mantadia National Park (AMNP)(18°23’ S latitude, 48°28’ E longitude) is located in the Moramanga region, and is one of the largest and most visited of Madagascar’s protected areas. The commune of Andasibe lies at its centre, and is surrounded by several villages to the north, whilst southerly villages are located along the Route Nationale 2.

#### Rainforest: Unprotected area

Situated in the Southeast region of Vatovavy-Fitovinany, the commune of Kianjavato (21°38’ S latitude, 47°87’ E longitude) is surrounded by several unprotected, and fragmented primary forests of Sangasanga, Tsiazonamboa, Ambatovaky, Tsitola and Vatovavy. These few remaining forests are characteristically low elevation humid evergreens surrounded by grassland, agricultural and secondary forests [28]. This forest primarily covers mountain ridges, with no fragment larger than 10 km^2^.

### Sampling strategy and interview protocol

Malagasy field staff were trained to interview independently within each village. Before interviews, the research objectives were explained to the village president who gave permission for survey commencement. Interviewers fluent in Malagasy conducted surveys, and were assisted by a local villager fluent in the regional dialect and customs.

Villages were categorised as ‘small’ or ‘large’, roughly corresponding to villages less than or greater than 100 households. During surveys all households in small villages were visited. The head of each household was requested for interview and verbal consent acquired. If the individual was absent, a maximum of two further appointments were scheduled.

Within larger villages only a sample of households were interviewed. The area was divided into districts and sampled using the zig-zag transect method [29]. This method allows households not located on streets to be visited, ensuring the representative sampling of populous villages. Each interviewer was instructed to walk a zig-zag transect, sampling every second household. If the head of the household wasn’t present, or was unwilling to be interviewed, the next household was surveyed. The potential bias introduced by unwilling respondents was probably negligible, as fewer than 2.5% declined to participate.

### Questionnaire design

A pilot questionnaire was devised and trialled in ANP during 2013. The results of the pilot questionnaire were not used, however the feedback shaped its ultimate design.

The questionnaire was divided into four sections addressing respondents’: i) demographic and socioeconomic information; ii) livestock ownership history; iii) dietary patterns; and iv) conservation perceptions and experiences (English and Malagasy questionnaires, S1 and S2 Tables). To promote trust, participants remained anonymous [30], however they were asked to reveal baseline demographic and socioeconomic information, such as employment, education level, ethnicity, household family structure, and livelihood indicators (asset ownership). Section two recorded participants’ details of livestock ownership, specifically: i) breed and quantity; ii) use of protective measures (i.e. coops); iii) perceived causes of poultry mortality (previous year and lifetime); iv) and attitudes towards fosas. In ultimately interpreting attitude as a driver of behaviour, we considered the social-psychological theory of planned behaviour [31], which proposes that behaviour is influenced by attitude, subjective norms, and perceived behavioural control [32]. This was considered when interpreting models of interviewee attitude.

Participants were directly asked sensitive questions regarding any fosa-conflict encounters, including: the number of fosa killed; seasonal and temporal occurrence; reason for killing; and their use of the fosa carcass. As a result, the collected data is potentially affected by non-response [33] and social desirability biases [34, 35]. The impact of asking sensitive questions in areas of varying sensitivity (i.e. protected versus unprotected areas) was illustrated by Razafimanahaka et al. [36] who reported higher rates of bushmeat consumption using sensitive questioning techniques in protected versus unprotected areas. This potential underreporting was considered when interpreting regional retaliatory killing estimates.

Given the interviewee’s potential reluctance to answer retaliatory killing questioning truthfully, interviewees were asked to report on other local villagers known to have killed a fosa. This questioning-method was included to produce a more accurate enumeration of fosas’ killed. Despite the potential for misinformation when incorporating third-party estimates, claims were validated through the crosschecking of spatial-temporal information, with interviewers able to deduce the year and location of each attack.

Ethical clearance and research approval was granted by The University of Oxford’s Central University Research Ethics Committee (Number: SSD/CUREC1A/14-010).

### Data analysis

Data were analysed using the statistical package R [37]. Fisher Exact Tests and Chi-Squared tests of Independence were used in the analysis of seasonality, diel period, forest type, and interviewee coop usage’s relationship with fosa poultry predation.

Three sets of generalised linear mixed effect models (GLMM) were used to examine the association of our explanatory variables with three response variables: i) fosas’ predation of poultry; ii) interviewees’ attitude towards fosas; and iii) retaliatory killing of fosas (S3 Table).

‘Last Year Predation’ was measured as a binary ‘yes/no’ response to a question asking if the respondent had experienced fosa poultry depredation during the previous year. Eight a priori variables thought likely to affect a household’s susceptibility to fosa predation, included; Forest Size, Village Size, River Barrier, Household Distance, Flock Size, Flock Coop, and Snare (S3 Table). Any continuous variables were standardised.

Region was included as a fixed blocking factor accounting for geographic variation. This was included in all statistical models as an aspect of study design. Given the nesting of households within villages the identities of individual villages were included as levels of a random effect. Pearson’s Chi-squared tests of Independence and Pearson Correlation Coefficients were used to identify collinearity among predictors. Forest type and Protection were not included as predictors in the GLMMs as they were highly confounded with Region.

A GLMM model with a binomial family and a logit link function (package lmer 1.1–12) was used to model the binary response variable, Last Year Predation. Akaike’s information criterion (AIC) was used to rank models according to the most parsimonious set of predictors [38]. Model averaging was not used for the reasons given in [39], principally that with any non-trivial level of collinearity among predictors, inevitable with most observational studies, model averaged parameter estimates become unreliable. Instead, where no single model was clearly dominant, we scrutinised the pattern of parameter estimates in the models with the highest weights. Where a complex model was judged not to have a meaningful improvement in model fit, we preferred a simpler model nested within if where the delta AICc value was small (<2.0).

Attitude towards fosas, measured on a 5-point likert scale was used as an ordinal response variable and modelled with the fixed-effect predictors, Poverty, Education, Poultry Owned, Lifetime predation, Conservation Benefit, Conservation Experience, and Conservation Attitude (and Village identity as a random effect). A cumulative logit function was fit using the R package ‘ordinal’ (version 2015.6–28). Model selection was as previously described.

The final series of models exploring the potential predictors influencing the retaliatory killing of fosas followed model one’s methods. The binary response variable (did interviewee’s report having killed at least one fosa during their lifetime) was thought to be influenced by Poverty, Conservation Experience, Conservation Attitude, Conservation Benefit, and Fosa Attitude (S3 Table). The household’s relative poverty level was estimated using several of the indicators contained within the Multidimensional Poverty Index (MPI) [40]. Unfortunately given the structure of our questionnaire our estimate contained only indicators from the MPI’s living standard dimension (excluding the health and education indicators). Our poverty estimate equally weighted six indicators to create a relative estimate of household poverty measuring poverty in an increasing scale from 0 to 1. Our indicators included assets ownership (i.e. mobile phone, bicycle), roof material, wall material, floor material, access to electricity and access to safe drinking water. Unfortunately, the poverty metric was unavailable for one of the regions (Boeny) due to changes in the content of the questionnaire between survey years. Consequently, two series of models were used for this response, with Poverty not included where all regions were used and with Poverty included (excluding observations from Boeny).

## Results

In total, 1746 households were interviewed (1325 male interviewees, 410 female interviewees, mean age 42.4 ± 13.3 and 38.8 ± 13.9) over six months from April–June 2014, and May–August 2015 (Table 1). 725 households were interviewed within and surrounding national parks, with 456 and 565 conducted within reserves and unprotected forests. Respondents were overwhelmingly farmers (77.5%). Most respondents’ highest level of educational attainment (Malagasy equivalent in brackets) was either primary school (EPP) (43.6%), or junior secondary (CEG) (24.6%). A substantial minority reported having no education (22.5%). Senior secondary (Lyceé publics) or tertiary (Université) education was not commonly reported (8.7% and 0.7% respectively).

### Livestock ownership and practices

Approximately 80% (n = 1391) of respondents owned livestock. Of these respondents, chickens (45.6%), zebu (25.1%) and ducks (15.6%) were most commonly owned. Of all the reared livestock, chickens were also bred in the greatest numbers, with a mean of 15.4 ± 15.63 (ranging from 1 to 150) owned per respondent.

87% of respondents who reared poultry used a protective structure. The majority, n = 595 (63%) stored their poultry in coops, inside their home (22%), or inside a cattle-pen (8.8%). 85% of poultry owners housed their poultry during the afternoon and evening, with the overwhelming majority doing so throughout the entire year (91%). Poultry were most commonly protected due to general security concerns and fear of predators (45.3% and 44.6% of respondents).

### Poultry mortality

86.7% of poultry owners (n = 1136) reported experiencing poultry mortality (to causes other than for consumption) during the last year. Mortality resulting from disease (40.4%) was most commonly reported. Mortality to predation was most often attributed to birds (17.0%) with fosa depredation recorded by 276 respondents (15.3%).

Predation by fosas was significantly more prevalent in western deciduous forests than the eastern rainforests, where 85.6% of predation was reported (Chi-squared test of independence, X^2^ = 121.67, df = 1, p < 0.01) (S4 Table).

Fosa depredation was significantly more likely to be reported as occurring during the evening in the dry season than any other season, and most commonly cited during the months of October to November (Chi-squared test of independence, X^2^ = 138.12, df = 4, p < 0.001) (S1 Fig). There was no evidence that the use of a coop was associated with the frequency of poultry depredation (S4 Table). In response to fosa depredation the majority of respondents reported: doing nothing (37.68%); improving their coop (36.52%); breeding dogs (8.12%); creating traps (4.35%); scaring the predator away (4.35%); or attacking the fosa (2.03%).

**Fig 2:**
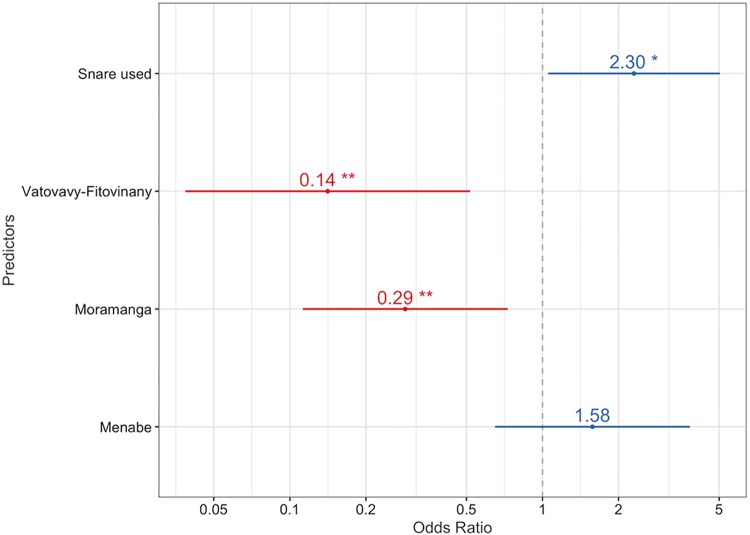
The significant predictors of fosas’ reported predation of poultry (with 95% confidence intervals). The x-axis displays the odds of households sustaining poultry predation that were located in a region or used a snare, against Boeny the reference region. The level of significance is denominated by * p < 0.05, and ** 0 < 0.01.

The most parsimonious model evaluating a household’s susceptibility to previous year poultry depredation included Region and Snare use (as the only significant predictors (at p < 0.01 and p < 0.05)(S5 Table). Odds ratios indicated that households in Moramanga were 3.45 times and Vatovavy-Fitovinany were 7.14 times less likely to report predation (Fig 2). The percentages reporting fosa predation were 22% in Boeny, 39% in Menabe, 11% in Moramanga and 7% in Vatovavy (Table 1). Poultry keepers who used snares were 2.30 times more likely to have sustained poultry predation than those who did not (Fig 2).

### Attitudes

**Fig 3:**
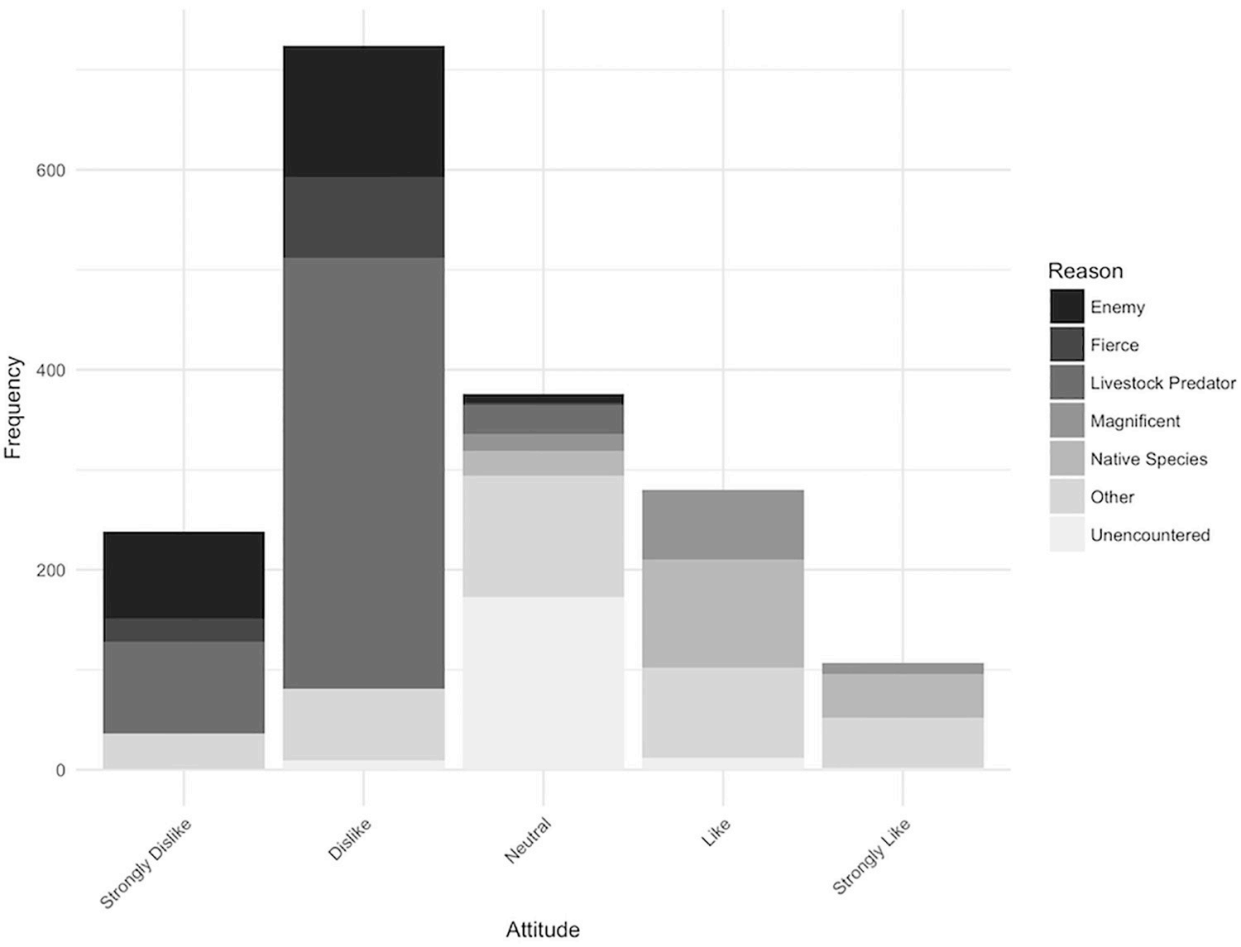
Interviewees’ stated attitude towards fosas and their reported reason.

Over half (55.7%, n = 967) of respondents disliked fosas (responding either ‘Strongly Dislike’ or ‘Dislike’)(Fig 3). The most commonly stated reasons were fosas’ depredation of poultry, their perception as their enemy and fosas’ reputedly fierce nature. Conversely, of the 22.4% of respondents that liked fosas (‘Like’ or ‘Strongly Like’), the principal reason was their status as a native species. Other respondents reported mixed-emotions because of their dislike of fosas’ poultry depredation but appreciation for their role in consuming pests such as rats.

74.4% (n = 1203) of respondents believed fosas were of no personal benefit, mostly citing poultry depredation (44.8%)(S6 Table). In contrast, the remaining respondents (n = 414) saw fosas as beneficial for their role as a rat predator (23.9%), source of tourism revenue (21.2%) and ecosystem component (16.5%). When asked if respondents were fearful for their poultry, the majority of respondents who kept poultry (52.6%, n = 713) weren’t concerned about fosas as a threat. The most popular reason stated was the presence of a geographical barrier (i.e. a river) surrounding their village (33%)(S6 Table). The majority (73%, n = 970) of respondents did not believe that fosa populations should be controlled, mostly because of their belief that fosas are one of god’s creations (33.3%). The remaining interviewees believed that fosa overpopulation increased poultry depredation (44.6%).

**Fig 4:**
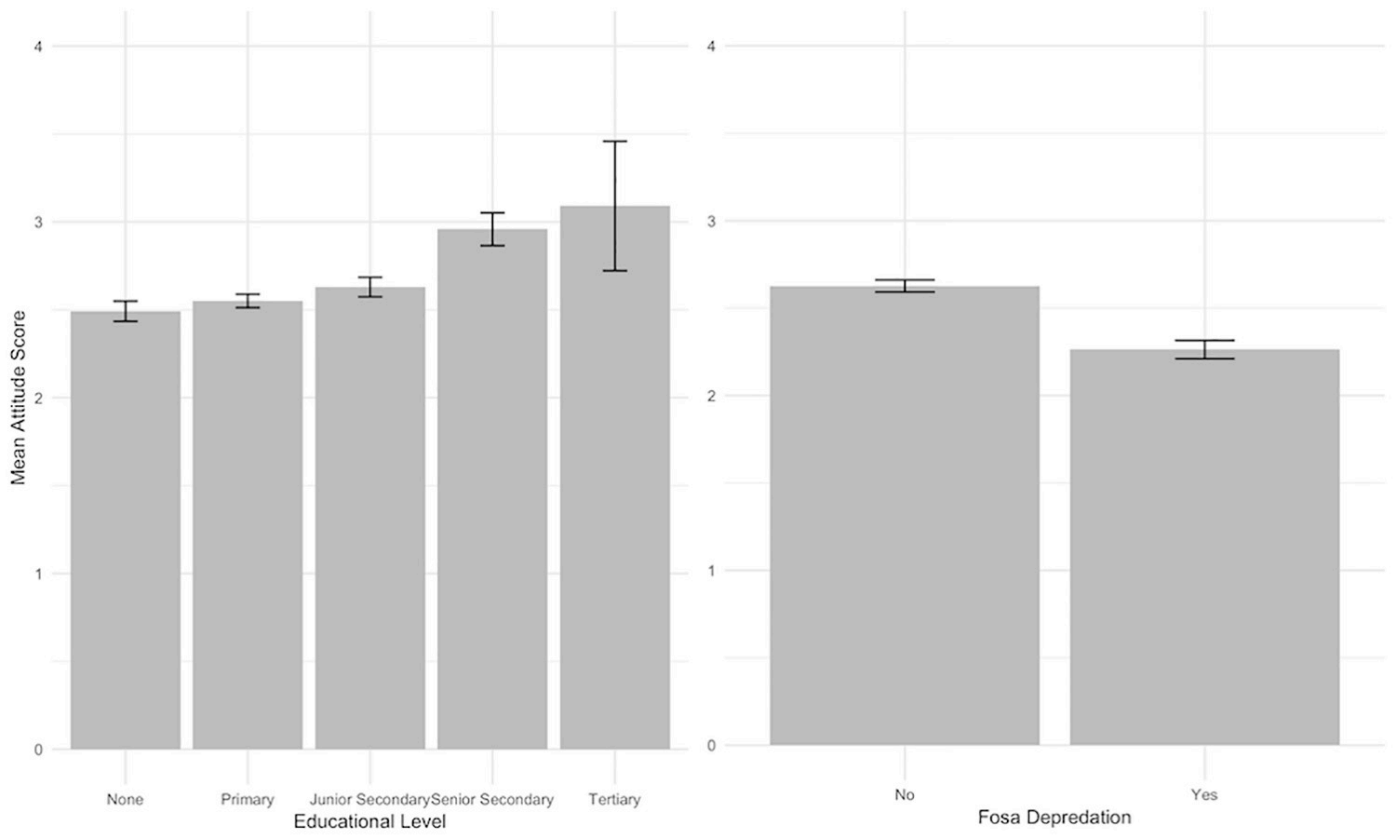
The significant predictors of interviewees’ stated attitude towards fosas (y-axis scale likert-scale from 1 strongly dislike to 5 strongly like), including the standard error of the mean.

The models examining interviewee attitude towards fosas revealed the strongest evidence for associations with Education, Lifetime Predation, and Region (S7 Table). Interviewees who have experienced fosa predation during their lifetime were significantly more likely to have a negative attitude (Fig 4). Likewise, interviewees’ with lower levels of educational attainment were also more prone to exhibit negative attitudes (Fig 4). Interviewees from Moramanga and Vatovavy-Fitovinany were more likely to hold a negative view of fosas compared with those from Menabe.

### Retaliatory killing

Only 41 respondents (2.3%) claimed to have attempted to kill a fosa during their lifetime, with 32% (n = 13) of these purportedly successful. A total of nine fosas were directly killed by interviewees during the previous year (with nine more killed during their lifetime). The vast majority (85%) of these 18 mortalities occurred in deciduous forests (Chi-sq x^2^ = 8, df = 1, p < 0.05, testing for a significant difference in retaliatory killing in deciduous versus rainforests). 52.5% of respondents used snares, or batons and blowpipes (12.5% respectively) to attack fosas. 85% of interviewees who reported attacking fosas cited poultry depredation as their reason. Approximately half of respondents either ate or discarded the fosa, with the remaining interviewees selling or giving the carcass as a gift.

Eighty-five (4.86%) respondents knew of someone who had attacked a fosa during their lifetime, with 63.5% of these reported attacks successfully killing the fosa. Twenty-one fosas were purportedly killed in the previous year, with approximately 50 killed during the lifetime of the respondents.

**Fig 5:**
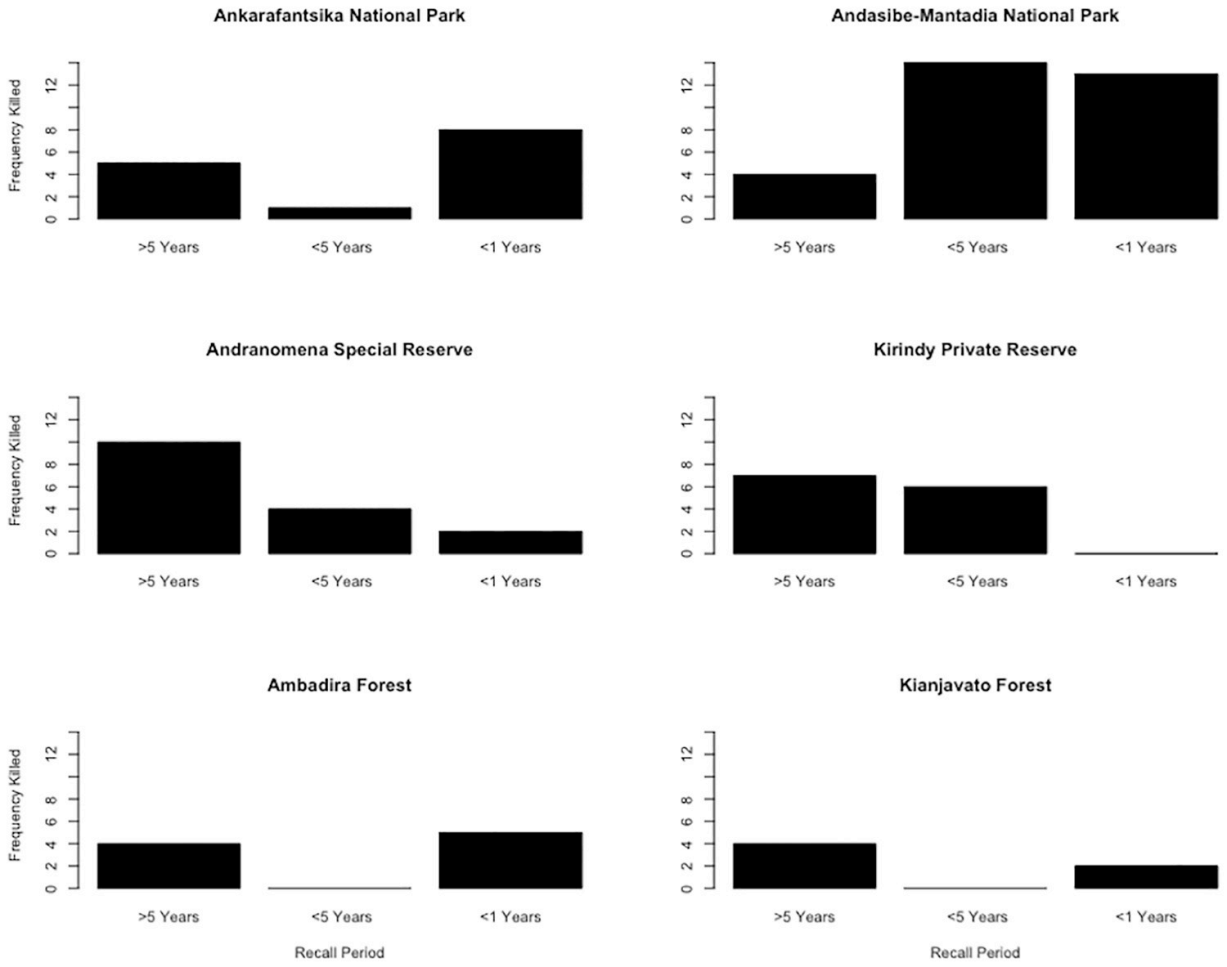
Interviewee estimates of the total fosas killed over the past year, five years and lifetime (each row displays national parks, reserves and unprotected forests).

Combining estimates of fosas killed directly by interviewees, and those reportedly killed by other local villagers, 30 fosas in total were killed during the previous year. A further 25 fosas were known to have been killed during the five preceding years, with 34 killed during the remainder of their lifetime (Fig 5).

AMNP (Moramanga) had the highest number of fosas killed during the last year (13 individuals) and last five years (14 individuals)(Fig 5). ANP (Boeny) had the second highest number of individuals killed (8) during the previous year. Across the regions, 4% of households killed fosas in Boeny, 2% in Menabe, 4% in Moramanga, and 0% in Vatovavy-Fitovinany. No patterns were discernible between forests of different type or protection level.

The final set of models investigated a household’s potential for retaliatory killing of fosas. The highest weighted model contained attitude and region, with interviewees’ more likely to retaliate when holding a negative attitude. The analysis was repeated, this time including Poverty and excluding ANP households (where this variable was not available); this yielded similar results with Attitude and Region, and the Poverty predictor to be contained in the most parsimonious model (S8 Table).

**Fig 6:**
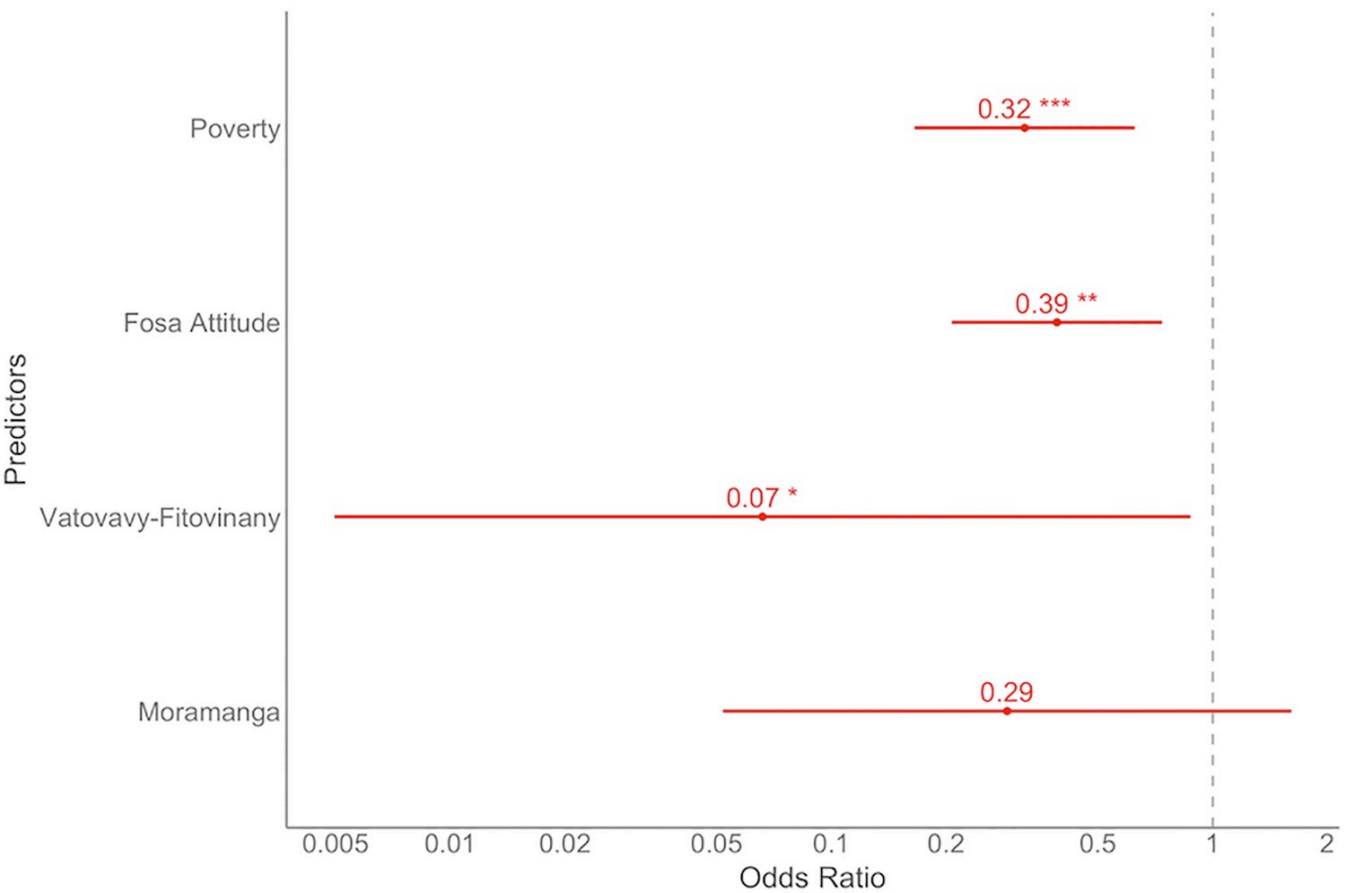
Forest plot of the three significant predictors of retaliatory killing, attitude, poverty and region (reference level is Menabe) (with 95% confidence intervals). The level of significance is denoted by *** p < 0.01, ** p < 0.05 and *** p < 0.10.

Scrutinising the final model (including Attitude, Region and Poverty) revealed that interviewees’ from Vatovavy-Fitovinany were less likely to engage in retaliatory killing, whilst those that held negative attitudes towards fosas, and/or wealthier respondents were significantly more likely to engage in retaliatory killing (S8 Table)(Fig 6). Odds ratios showed poorer interviewees’ were 3.13 times less likely to kill a fosa (an increment of 1.0 on the ‘Poverty’ scale, i.e. the difference between the poorest and wealthiest individuals, had this effect on the response), and interviewees’ who held favourable views towards fosas were 2.56 times less likely to have attempted killing them (an increment of one unit on this ordinal scale had on average this effect on the response, Fig 6).

## Discussion

Fosa predation was reported by interviewees to be one of the greatest causes of poultry mortality. Its occurrence was concentrated in the western forests, and most often recorded during the dry season in the evening. There was no evidence that poultry coops were effective in reducing fosa predation, likely owing to their poor construction. Most interviewees’ disliked fosas, with poultry predation the most commonly stated reason. Respondents who said they liked fosas commonly cited their endemicity, their impressiveness and their role as a rat predator. Interviewees that had suffered poultry depredation and those with less education were most likely to dislike fosas. Interviewees that disliked fosas and those that were wealthy were most likely to have killed a fosa. We estimated that a minimum of thirty fosas was killed in retaliation across our study regions during the year before interviews. Our results suggest that a strategy to reduce retaliatory killing should focus upon educating communities of fosas’ ecological importance, whilst promoting the construction of sustainably built, robust poultry coops, in align with the use of guard dogs. Vaccination programmes should also be considered within this strategy to reduce the economic cost of the greatest cause of poultry mortality, disease, and to reduce the risk of greater disease transmission if poultry are housed within coops.

### Poultry depredation

Recent estimates from Ranomafana suggested that < 1% of households’ poultry mortality was attributable to fosas [21], supporting historical reports of anecdotally infrequent attacks [9, 18]. By contrast, approximately one in six interviewees’ in our study reported fosa depredation. Together, these observations indicate that perceived predation by fosas is, while not rare, not the most economically damaging agent. Disease was reported to have killed nearly 40% of interviewees’ poultry, with Newcastle disease the likely virus. Newcastle disease has been previously reported as the principal cause of rural poultry mortality in other areas of Madagascar [41], and it is undoubtedly the greatest cause of economic loss for rural poultry owners. Mortality from birds of prey was the second most common cause, although its impact is less economically detrimental due to their predation of chicks (costing around 400 Ariary at current prices). Conversely, fosas commonly consume adult chickens, typically valued at around 12,000 Ar (3,600 Ar = 1 USD). Fosas’ tendency to kill all chickens when inside a coop, a predation behavioural characteristic repeatedly retold during interviews, and previously acknowledged in the literature, can result in a substantial cost to affected households (Albignac, 1973; Merson personal observ.). With some 70% of Malagasy households currently living below the poverty line (< 1 USD/day), the individual cost of fosas’ predation upon respondents’ livelihoods may be significant, despite being potentially half the economic cost of disease. In the absence of structurally robust coops effective at reducing wildlife predation, retaliatory killing of nuisance fosas is the likely response.

Fosas’ predation of poultry was more frequent in deciduous forested regions. This may be attributable to higher fosa densities in deciduous forests [14, 15]. Anecdotal reports also support these estimates, with considerably higher trapping success in deciduous regions (Luke Dollar, Merson personal observ.). Higher fosa predation rates may also be due to lower prey availability in accord with greater aestivation of deciduous forest living species during the dry season.

Throughout both forest types, poultry depredation was significantly more likely to occur during the dry season (May to November). This pattern aligns with the aestivation and torpor of many of Madagascar’s mammals [42–44]. Hawkins and Racey’s [12] dietary analysis found seasonal differences in the relative frequency (but not absolute number) of prey species in the fosas’ diet. It might then be possible that higher winter poultry predation is a preferred strategy to combat reductions in species common during the summer months, such as tenrec [45]. Furthermore, most respondents claimed fosa poultry predation occurred most frequently between October and November. This is the end of the dry season, when fosas would have felt the brunt of winter and its associated reductions in prey, but also coincide with fosas’ mating season. A recent study of fosa sociobiology in western Madagascar estimated male home ranges to have increased during the breeding season from c. 40 to c. 50 km^2^ [46]. These changes in male home range behaviour, including the likelihood of poorer body condition and increased metabolic demands, may create a propensity for greater overlap between fosas and human settlements.

Lastly, fosa predation of poultry was significantly more prevalent during the evening. Fosas’ activity may be largely cathemeral, but it is also known to peak during the crepuscular hours [47, 48], with high activity during the evenings [49, 50]. Recent research revealed fosas may alter their activity pattern to avoid humans and dogs [48–50], It may then be expected, similar to other instances of carnivore livestock predation [51], that fosas would choose to hunt poultry when human activity is presumably at its lowest during the evenings.

Households that used snares and those living in Menabe were significantly more likely to experience fosa predation. The positive association with snare use is potentially explained by the proximate reason that households using snares have experienced higher historic levels of fosa predation. Likewise, Menabe is a deciduous forest and contains Kirindy Reserve, one of the few forests in Madagascar with a highly habituated fosa population. Fosas in this sub-population may be less deterred by human presence, and more willing to venture into villages. In addition, this region continues to experience severe deforestation [27], with many of the remaining forests too small to sustain long-term fosa populations [14]. Given fosas’ large home ranges, individuals in this region may be more inclined to leave the forest, promoting interaction with nearby villages.

### Attitudes

The results of the GLMM estimated fosa poultry predation to be strongly linked to respondent attitudes, with interviewees who reported poultry losses to fosas significantly more likely to dislike them. This aligned with most interviewees’ cited qualitative reason for disliking fosas (poultry predation). Despite seemingly pragmatic reasons for Malagasy dislike of fosas, underlying *fady* may also have shaped modern perceptions. Fosas’ similarity to dogs was frequently mentioned, with dogs disliked both for cultural perceptions as being ‘dirty’, and for their occasional theft of food. Another common *fady* regularly cited was their dislike of fosas due to fosas’ consumption of their ancestor’s remains. Given some burial sites (for example tombs within mountainside caves) could be easily accessed by foraging fosas, the claim may have some validity and has been reported elsewhere [19, 23].

Other commonly cited anecdotes included stories of fosas having eaten missing people, packs of fosas attacking and consuming zebu, and attacks upon lone individual villagers–often resulting in death. The validity of these stories is unknown, however, given our understanding of fosas’ behaviour, they are unlikely to be true. These stories do however reinforce a fundamentally negative perception of fosas. Our observation that attitudes were related to education suggests this affect could be reduced through educational programs.

Fosas were commonly liked as a predator of rats, a native species, and interestingly appreciated for their aesthetic appeal. Educational programs highlighting fosas’ benefit as a rat predator were initiated by co-author L. Dollar in ANP wherein almost half of the total respondents that cited this reason were located.

Interviewees’ who had experienced fosa predation, and those with lower education were more likely to negatively perceive fosas. Education is more likely to expose individuals to concepts of conservation, and its benefits. Consequently, fosas may be perceived not only as a pest, but also as an important component of Madagascar’s ecosystem. Interestingly, interviewees’ from Moramanga were most likely to negatively perceive fosas. This region encompassed AMNP, Madagascar’s most visited national park. Despite its monetary benefits (employment, tourist revenue, park fees), many of the households interviewed were within the poorest villages. These villagers are still reliant upon traditional livelihoods, and are typically not benefitting from the park. It is also probably relevant that this region also recorded the greatest number of fosas killed, highlighting the association between attitude and retaliatory killing.

### Retaliatory killing

In the previous year, nine fosas were reportedly killed by interviewees, increasing to thirty when including third-party accounts. This estimate should be interpreted as a minimum given the question’s sensitivity, and the interviewee’s probable reluctance to answer questions truthfully for fear of persecution by law enforcement or Madagascar National Park officials. Given fosas in Madagascar are largely known to be a protected species, interviewees are unlikely to reveal their killing when directly asked. The reluctance to answer honestly when directly questioned about illegal or socially unacceptable behaviours has been demonstrated for direct questioning of bushmeat hunting in Madagascar [36], and underreporting of retaliatory killing of fosas in our study is undoubtedly an issue.

Interpreting the impact of fosa mortalities is difficult, with island-wide population estimates likely inaccurate, and varying between 2,635–8,626 adults [52]. However, most of these sub-populations persist in extremely fragmented forests, thought to be too small to maintain long-term populations [14]. Hence, the fosa population is reportedly decreasing [53]. For some isolated populations, this human persecution can only accelerate their decline. It’s therefore possible that these mortality rates are unsustainable, particularly within smaller forests.

In absolute numbers AMNP and ANP, the two largest forests and national parks, had the highest number of fosas killed during the previous year (including total recorded). Despite offering the highest legal protection, this did not demonstrably reduce retaliatory killing compared to forests of less protection. It could also be assumed that underreporting in these regions would be at its highest, as increased forest protection, NGO, and tourism presence, would likely result in greater sensitivity to report illegal or socially undesirable behaviours [36].

Respondents’ with negative attitudes towards fosas were more likely to kill them if they had reported poultry predation. While this link is intuitively rational, the socio-psychological theory of planned behaviour suggest that attitudes alone, do not always predict behaviour, with cultural factors typically an important influence [54]. Carnivore killing results from an interplay between social and cultural factors, in addition to the individual–effective strategies for prevention need to take all of these into account [55]. Contrary to our expectation, wealthier households were most likely to have killed fosas. It was thought that poorer households with lower food security would be less tolerant of fosa predation and most likely to retaliate. We speculate that these households may have political or social power in association with greater wealth, and feel at greater liberty to kill fosas than poorer households with presumed less social/political power. Future research into community political and social structure may shed light on this.

When asked what interviewees’ did with a killed fosas’ remains, approximately half of respondents said they consumed it, further supporting the widespread consumption of fosa [50, 56–58]. This trend likely reflects growing poverty and food insecurity across Madagascar.

Fosas were principally killed in retaliation for their consumption of poultry, likely underpinned by rational human’s inclination to protect their livelihoods. However, it is also likely to be exacerbated by the unclear legislation defining fosa protection [16]. While nominally protected under national law the killing of nuisance species that damage an individual’s property is permitted (Category 1, Class 2 Protected Species). Such conflicting legislation is counterproductive to efforts to protect fosas, and to establish an underlying protective culture towards Madagascar’s wildlife.

### Conclusions

Our results suggest that efforts to promote knowledge of the fosa could encourage tolerance, even in the presence of some impact on livelihood. Fosa predation was reported by a minority of poultry keepers, where it was likely not as economically important compared with the major source of mortality, disease. Educational material has potential to promote positive attitudes based on fosas’ status as an iconic endemic species, tourist attraction and predator of other pests, including rats. Furthermore, the design and creation of robust poultry coops, in align with the use of guard dogs, an observed spatio-temporal deterrent of fosas [48–50, 52, 58] could reduce retaliatory killing. Disease vaccination programmes should be considered with the implementation of robust coops and guard dogs to reduce its economic cost, and the risk of greater disease transmission amongst coop living chickens. Given the island-wide intrusion of humans into forested habitat, retaliatory killing reduction strategies must be trialled and deployed to ensure the persistence of one of Madagascar’s most important species.

## Supporting information

S1 Fig Fosa poultry predation by season and diel period (Chi-squared test of independence, X^2^ = 138.12, df = 4, p < 0.001). (DOCX)

S1 Table English questionnaire translation of Malagasy questionnaire used during interviews. (DOCX)

S2 Table Malagasy questionnaire used during interviews. (DOCX)

S3 Table Description of the response variables and predictors used within each of the three sets of GLMMs. The model variables included: Dependent Variable 1/2/3 (DV1, DV2, DV3); independent variable model 1/2/3 (IV1, IV2, IV3); Fixed Blocking Factor model 1/2/3 (BF1, BF2, BF3), and random effect model 1/2/3 (RE1, RE2, RE3).
(DOCX)

S4 Table a) The total number of households that own poultry in deciduous and rainforests to have experienced fosa depredation (percentage of households in brackets) (Chi-squared test of independence, X^2^ = 121.67, df = 1, p < 0.01). b) The total number of households that own poultry to have experienced fosa depredation that house their poultry in a coop and not in a coop (percentage of households in brackets) (Chi-sq X^2^ = 0.69786, df = 1, p = 0.4035).
(DOCX)

S5 Table a) Model selection output for the highest weighed models of the predictors of a households’ likelihood to sustain fosa poultry predation. Preferred model is in bold. Degrees of freedom (df), log likelihood (logLik), Akaike’s Information Criterion (AIC_c_), relative change in Akaike’s Information Criterion from top model (ΔAICc), and Akaike’s Information Criterion weight (AIC_c_wt). b) The modelled output for the most parsimonious predictors, Snare and Region. P-value (Pr (>|z|)) at significance level (p < 0.001***, p < 0.01 **, p < 0.05 *).
(DOCX)

S6 Table Interviewees’ stated response and reason to various attitudinal questions. (DOCX)

S7 Table a) Model selection output for the highest weighed models containing the predictors of a households’ attitude towards fosas. Preferred model is in bold. Degrees of freedom (df), log likelihood (logLik), Akaike’s Information Criterion (AIC_c_), relative change in Akaike’s Information Criterion from top model (ΔAICc), and Akaike’s Information Criterion weight (AIC_c_wt). b) The modelled output for the most parsimonious predictors, Education, Lifetime Predation and Region. P-value (Pr (>|z|)) at significance level (p < 0.001***, p < 0.01 **, p < 0.05 *).
(DOCX)

S8 Table a) Model selection output for the highest weighed models containing the predictors of a households’ retaliatory killing of a fosa. Preferred model is in bold. Degrees of freedom (df), log likelihood (logLik), Akaike’s Information Criterion (AIC_c_), relative change in Akaike’s Information Criterion from top model (ΔAICc), and Akaike’s Information Criterion weight (AIC_c_wt). b) The modelled output for the most parsimonious predictors, Fosa Attitude, Poverty and Region. P-value (Pr (>|z|)) at significance level (p < 0.001***, p < 0.01 **, p < 0.05 *).
(DOCX)
