# Supplementary figures and images for: Retaliatory killing and human perceptions of Madagascar’s largest carnivore and livestock predator, the fosa (Cryptoprocta ferox)
Source: PLoS One. 2019 Mar 15;14(3):e0213341. doi: 10.1371/journal.pone.0213341 (PMC6420034; doi:10.1371/journal.pone.0213341)

**S1 Fig.** Poultry predation season versus diel period. (Chi-squared test of independence, X2 = 138.12, df = 4, p < 0.001).


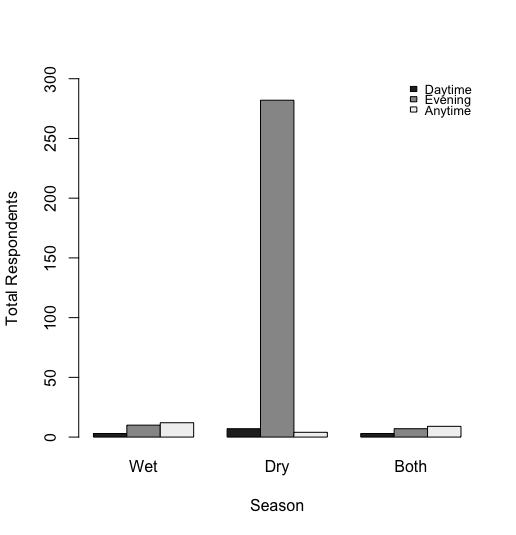

Supplement: S1 Fig — (DOCX) [file pone.0213341.s001.docx]
