# Supplementary material for: Retaliatory killing and human perceptions of Madagascar’s largest carnivore and livestock predator, the fosa (Cryptoprocta ferox)
Source: PLoS One. 2019 Mar 15;14(3):e0213341. doi: 10.1371/journal.pone.0213341 (PMC6420034; doi:10.1371/journal.pone.0213341)
Supplement: S1 Table — (DOCX) [file pone.0213341.s002.docx]

**S1 Table.** English questionnaire translation of Malagasy questionnaire used during interviews.

**Human-Wildlife Relationship Questionnaire**  Interviewer Name:

**Please read to participants the study information and ensure verbal consent is given. Follow instructions in italicised Text. Please**

**complete entire questionnaire.**

**Part One: Demographic:** Q1. Respondent Information.

| Date: |  | Household # |  |
| --- | --- | --- | --- |
| Interview Start/Finish: |  | GPS S GPS E |  |
| Village |  | Sub-Village |  |
| Sex |  | Age (years) |  |
| Marital Status |  | Ethnicity |  |
| # People living in household >18yrs M/F |  | # Children (<18yrs) M/F |  |
| Land > 500M |  | * Household-head Education level |  |
| ** Pets |  | Religion |  |

* [*For Education ask if highest level is: none, primary, secondary or tertiary*] ** [*What type of pet, and how many?*]

Q2. Are you from this village originally? YES NO

If NO, where? ________________________________ How many years have you lived here? ______________

Household-head Job: _______________________ Number of children <18 in school? ____________________

Q3. Do you grow crops? YES* NO *If Yes, type:

1. _____________ 2. _____________ 3. _____________ 4. _____________ 5. _____________

Q4. Do you own an ox-cart? YES NO If so, how many? ______

Q5. What type of cooking material do you typically use? _______________________Q6. What kind of light do you use in your home? ___________________ Q7. How happy are you with this method in lighting your home?

VERY HAPPY QUITE HAPPY NEUTRAL QUITE UNHAPPY VERY UNHAPPY

Q8. How do you acquire your drinking/cooking water? _____________________________________________

Q9. Assets. ELECTRICITY [*If so, please circle assets owned*] TV RADIO FRIDGE

CAR MOTORCYCLE/SCOOTER BICYCLE WATCH MOBILE PHONE

Q10. How many rooms do you have in your household? _____ Q11. How many people sleep per room? ______

Roof Material [*observe*] RAFFIA METAL GRASS PALM OTHER: _______________

Wall Material [*observe*] MUD BAMBOO WOOD BRICK CEMENT-BLOCK

Floor Material [*observe*] EARTH TREE-BARK WOODEN-PLANK CEMENT CERAMIC TILES

**Part 2: Wildlife-livestock relationship**

Q1. If you own livestock, what breeds and how many do you own? *[If no skip to Q16*]

|  | Quantity | Protected Y/N? | How? |
| --- | --- | --- | --- |
| 1. Adult Chicken 2. Chick |  |  |  |
| Duck |  |  |  |
| Turkey |  |  |  |
| Goat |  |  |  |
| Pig |  |  |  |
| Zebu |  |  |  |
| Other |  |  |  |

Q2. Do you allow your poultry to ALWAYS roam freely? YES NO

*[If yes, skip to Q11. If no, please continue with the next question. Please tick multiple boxes if participant stores poultry in more than one place/time.]*

Q3. What poultry do you store? CHICKEN DUCK TURKEY OTHER: _________

Q4. Where do you store your poultry? COOP INSIDE-HOUSE OTHER: __________

Q4a. Ranking of coop quality (1 = Very weak, 5 = Very Strong)? ________ Photo number: ______________

Q5. What time of day do you store your poultry? MORNING MIDDAY AFTERNOON EVENING

Q6. What time of year do you store your poultry? DRY WET BOTH

Q7. Why do you store your poultry? ____________________________________________________________

__________________________________________________________________________________________

Q8. Do you set up snares to protect your poultry? YES NO [*If no, skip to Q11*]

Q8a. Where do you set up your snares? __________________________________________________________

__________________________________________________________________________________________

Q9. Have you ever found any animals in your snares? YES NO [*If yes, fill in box below*]

| What type of animal did you find? | How many times in your lifetime? | How many times since last year? | What time of year? (dry/wet/both) | ** What time of day? | What did you do with the animal? |
| --- | --- | --- | --- | --- | --- |
| Wild cat |  |  |  |  |  |
| Fosa |  |  |  |  |  |
| Small Indian civet |  |  |  |  |  |
|  |  |  |  |  |  |

[* *Since local event* ** *Morning/Midday/Afternoon/Evening*]

Q10. Has anyone else in your village ever found an animal caught in a snare protecting their poultry coup? YES NO [*If yes complete table below*]

| Who/where? [*use spatial/temporal cues*] | Did you see it? | What animal snared? | How many times in lifetime? | How many last year? | ** What time of day? | What time of year? (dry/wet/both) | What did they do with it? |
| --- | --- | --- | --- | --- | --- | --- | --- |
|  |  |  |  |  |  |  |  |
|  |  |  |  |  |  |  |  |
|  |  |  |  |  |  |  |  |
|  |  |  |  |  |  |  |  |

[* *Since local event* ** *Morning/Midday/Afternoon/Evening*]

Q11. Do you know of anyone whose livestock has been killed by a fosa? YES NO *[If yes continue, if no please skip to Q17]*

| Who/where? [*use spatial/temporal references*] | Did you see it? | Poultry breed? | Cost? | Lifetime | Number during last year? | What time of year? (dry/wet/both) | What time of day? | What did they do with it? | What did they do to prevent future attacks? |
| --- | --- | --- | --- | --- | --- | --- | --- | --- | --- |
|  |  |  |  |  |  |  |  |  |  |
|  |  |  |  |  |  |  |  |  |  |
|  |  |  |  |  |  |  |  |  |  |
|  |  |  |  |  |  |  |  |  |  |

[** *Morning/Midday/Afternoon/Evening*]

Q12. Do you know of anyone else who has ever hunted fosas in retaliation for killing their poultry or for any other reason? YES NO

Q12a. Did you see it? YES NO [*If No, please skip to Q14*].

| What killed your poultry? | What kind of poultry? | What was the cost of the animal? | How many killed over lifetime? | How many killed since last year? | * What time of year? | ** What time of day does this mostly occur? | Do you usually see it happen? | If so, what do you typically do? | What have you done to prevent it happening in the future? |
| --- | --- | --- | --- | --- | --- | --- | --- | --- | --- |
| Car |  |  |  |  |  |  |  |  |  |
| Bird of Prey |  |  |  |  |  |  |  |  |  |
| Fosa |  |  |  |  |  |  |  |  |  |
| Disease |  |  |  |  |  |  |  |  |  |
| Snake |  |  |  |  |  |  |  |  |  |
| Wild Cat |  |  |  |  |  |  |  |  |  |
|  |  |  |  |  |  |  |  |  |  |
|  |  |  |  |  |  |  |  |  |  |

Q12b. When? *Year* ____ ____ *Season* ____ ____ Q12c. Why did they kill it?_____________________________________________________________________________

Q12d. How many times? ____ *ID*: ____ Q12e: How did they hunt it? ___________________________________________________________________________________

Q13. Were they successful in hunting it? YES NO [*If No, please skip to Q14*] Q13a. If so, how many fosas have they killed? _______________

Q13b. Where? ______________ Q13c. What did they do with them? ___________________________________________________________________________________

Q14. Have any of your poultry ever disappeared? YES NO How many last year?_________ Have you seen any of your poultry being killed, if so what? [*Ask if the following killed their poultry and list any other, find out specifics of each*] *[If No please skip to Q16]*

* *Wet/Dry/Both* ***Morning/Midday/Afternoon/Evening*

Q15. Have you (or anyone in your household) ever hunted fosas in retaliation for killing your poultry or for any

other reason? YES NO [*If No, please skip to Q17*]

Q15a. When? *Year* ____ ____ *Season* ____ ____ Q15b. Why? _______________________________________

__________________________________________________________________________________________

Q15c. How many times? ____________ Q15d: How do you hunt fosas? _______________________________

__________________________________________________________________________________________

Q16. Were you (or anyone in your household) successful in hunting fosas? YES NO [*If No, skip to Q17*]

Q16a. If so, how many fosas have you killed? ____________ Q16b. Where? ___________________________

Q16c. What did you do with it? _______________________________________________________________

Q17. How do you feel about the fosa?

STRONGLY DISLIKE QUITE DISLIKE NEUTRAL QUITE LIKE STRONGLY LIKE

Q18. Why do you feel this way? _______________________________________________________________

__________________________________________________________________________________________

Q19. Does the fosa provide any benefit to you? YES NO What benefit/why no benefit?

__________________________________________________________________________________________

Q20. Does the fosa provide any benefit to the ecosystem? YES NO What benefit/why no benefit?

__________________________________________________________________________________________

Q21. Why do you think the fosa wants to eat your poultry? __________________________________________

Q22. Are you scared of the fosa for your own safety? YES NO Why? ____________________

__________________________________________________________________________________________

Q23. Are you scared of the fossa for your poultry? YES NO Why? ____________________

__________________________________________________________________________________________

Q24. Do you think the fosa’s population should be controlled? YES NO Why? ____________

__________________________________________________________________________________________

Q25. Do you believe in any taboos relating to the fosa? YES NO If so, what taboos? _________

__________________________________________________________________________________________

**Part 3: Diet**

Q1. What are your household’s five most consumed foods (could you please rank them in most eaten)?

1. _____________ 2. _____________ 3. _____________ 4. _____________ 5. _____________

Q2. Do you consume these foods seasonally or perennially? [*Please list*]

DRY:____________________________________________________________________________________

WET:____________________________________________________________________________________
BOTH:___________________________________________________________________________________

Q3. What are your household’s five most accompaniments to rice/maize (could you please rank them)?

1. _____________ 2. _____________ 3. _____________ 4. _____________ 5. _____________

Q4 What are your household’s five most consumed fruits (could you please rank them)?

1. _____________ 2. _____________ 3. _____________ 4. _____________ 5. _____________

Q5. What is your primary source of meat? DOMESTIC ANIMAL WILD ANIMAL

Q6. On average how many days per week does at least one meal contain meat? Dom ___ Wild ___ None __

Q7. Is there a period of the year in which you have don’t have enough food?

Season: _______ From: _______ To: _______ #Months:________

Q8. What is your favourite domestic meat? _______________ Why? _________________________________

Q8a. TEST. Which of those animals have you ever seen in the forest? _________________________________

Q8b. Please rank in order of preference (only ranking what you have eaten):

Zebu ____ Duck ____ Goat ____ Chicken ____ Turkey ____ Pig ____ Sheep: ____ Other: ________

Q8c. Why did you rank the animals in this order? _________________________________________________

_________________________________________________________________________________________

Q8d. Please rank in order of most consumed (only ranking what you have eaten):

Zebu ____ Duck ____ Goat ____ Chicken ____ Turkey ____ Pig ____ Sheep: _____ Other: ______

Q8e. Why do you choose to eat domestic meat? __________________________________________________

_________________________________________________________________________________________

­­­­Q9. Have you ever eaten any of these animals before during your lifetime? YES NO

[*If yes, please answer Q9a, If no please skip to Q12*]

Q9a. What is your favourite wild meat? _______________ Why? ____________________________________

Q9b. Please rank in order of preference (only ranking what you have eaten):

Lemur ____ Bird ____ Tenrec ____ Reptile ____ Pig ____ Carnivore ____ Aquatic ____ Bats ____

Q9c. Why did you rank the animals in this order? _________________________________________________

_________________________________________________________________________________________

Q9d. Why do you choose to eat wild meat? ______________________________________________________

_________________________________________________________________________________________

Q10. Please rank in order of preference (only ranking what you have eaten):

Lemur ____ Bird ____ Tenrec ____ Reptile ____ Pig ____ Carnivore ____ Aquatic ____

Bats ____ Zebu ____ Duck ____ Goat ____ Chicken ____ Turkey ____ Pig ____

Q11. In your lifetime have you ever eaten?

| Animal | Species | How many times eaten during lifetime? | How many times during previous year? | How many times in the last seven days? | Which season do you most commonly eat (wet/dry/both)? | How did you acquire it?  (Bought/Hunt/Gift) | Where Bought/Hunted and/or who gave? | If bought, cost per kg or animal? | Why buy (cheap/tasty?) /hunt (i.e. opportunistic, specific) /gift (special occasion)? | How hunted? |
| --- | --- | --- | --- | --- | --- | --- | --- | --- | --- | --- |
|  |  |  |  |  |  |  |  |  |  |  |
|  |  |  |  |  |  |  |  |  |  |  |
|  |  |  |  |  |  |  |  |  |  |  |
|  |  |  |  |  |  |  |  |  |  |  |
|  |  |  |  |  |  |  |  |  |  |  |
|  |  |  |  |  |  |  |  |  |  |  |
|  |  |  |  |  |  |  |  |  |  |  |

Q12. Have you ever hunted and sold?

| Animal | Species | How many times during lifetime | How many times during last year? | How many times on average do you hunt per week? | How do you hunt it? | Which season do you most commonly sell? | On average how many individuals do you catch per hunt? | Where do you hunt it? | Where do you sell it? | How much do you sell it for per kg/animal? | Why do you hunt and sell? |
| --- | --- | --- | --- | --- | --- | --- | --- | --- | --- | --- | --- |
|  |  |  |  |  |  |  |  |  |  |  |  |
|  |  |  |  |  |  |  |  |  |  |  |  |
|  |  |  |  |  |  |  |  |  |  |  |  |

Q13. What type of wild animals have you tried to hunt for most in your life?

___________________________________________________________________________________

Q13a. Please rank in order hunting success (only animals that you have hunted).

Lemur __ Bird __ Tenrec __ Reptile __ Pig __ Carnivore __ Aquatic __ Bats __

Q13b. What animal was easiest to catch? _______________ Why? ____________________________

___________________________________________________________________________________

Q14. Are there any taboos relating to any wild animals in this area? YES NO

What?_____________________________________________________________________________

**Part 4. Wildlife conservation**

Q1. What do you think wildlife conservation is? ___________________________________________

__________________________________________________________________________________

Q2. Why would we conserve wildlife? ___________________________________________________

__________________________________________________________________________________

Q3. Who do we protect wildlife for? __________________ Why do you feel this way? ____________

__________________________________________________________________________________

Q4. Do you like wildlife conservation?

STRONGLY LIKE QUITE LIKE NEUTRAL QUITE LIKE STRONGLY LIKE

Q4a. Why do you feel this way? _______________________________________________________

__________________________________________________________________________________

Q4b. Do you receive any benefit from conservation? YES NO

What?_____________________________________________________________________________

Q5. Do you know that this is a protected (or unprotected) area? YES NO

Q5a. What does this mean? ____________________________________________________________

Q6. Why do you think the forest was protected? ____________________________________________

___________________________________________________________________________________

Q7. How do you feel about this forest being established as a protected (or unprotected) area?

STRONGLY LIKE QUITE LIKE NEUTRAL QUITE LIKE STRONGLY LIKE

Q7a. Why do you feel this way? ________________________________________________________

Q7b. Do you receive any benefit from this forest? YES NO

What? _____________________________________________________________________________

Q8. Has anyone ever spoken to you about conservation? YES NO

How many times? ______ Job? _____________ Who did they work for? ________________________

Where were they from? _______________________________________________________________

What did they tell you? _______________________________________________________________

___________________________________________________________________________________

Did this change your perception of wildlife conservation? YES NO

WHY?_____________________________________________________________________________
