# Supplementary material for: Retaliatory killing and human perceptions of Madagascar’s largest carnivore and livestock predator, the fosa (Cryptoprocta ferox)
Source: PLoS One. 2019 Mar 15;14(3):e0213341. doi: 10.1371/journal.pone.0213341 (PMC6420034; doi:10.1371/journal.pone.0213341)
Supplement: S2 Table — (DOCX) [file pone.0213341.s003.docx]

**S2 Table.** Malagasy questionnaire used during interviews.

**Fanontaniana Fanadihadiana momba ny fifandraisan’ny olombelona sy ny zavaboahary**

Anaran’ny mpanadihady:

Vakio amin’ny olona hadihadianao ity alohan’ny hametrahanao fanontaniana aminy ary anontanio raha manaiky ny handray anjara izy. * Araho tsara ny fanoroana voasoratra toy ny soratanana . Vitao hatramin’ny farany ny fanadihadiana.

**Fizarana 1: Filazana ankapobeny:** Q1. Momba ny hadihadiana:

| Daty: |  | Laharan’ny tokantrano# |  |
| --- | --- | --- | --- |
| Ora fanombohan’ny fanadihadiana/ famaranana: |  | GPS S GPS E |  |
| Fokontany |  | Tanàna |  |
| Lahy sa Vavy |  | Taona |  |
| Manambady sa tsia/Maty vady sns.. |  | Foko |  |
| # Isan’olona isan-tokantrano >18 taonz. #Lehilahy/Vehivavy |  | # Ankizy (< 18 taona) #Lehilahy/Vehivavy |  |
| Tany > 500m2 |  | * Fari-pianaran’ny loham-pianakaviana |  |
| ** isan’ny biby |  | Finoana |  |

* [*tsy nianatra, ambaratongs voalohany, faharoa, fahatelo, ambony*] ** [*karazam-biby, firy?*]

Q2. Teraka teto ve ianao? ENY TSIA

Raha tsia, avy aiza? ______________________________ Hafiriana ianao no nipetraka teto? __________________________

Asan’ny filoham-pianakaviana: _____________________ Isan’ny ankizy mianatra <18 taona i?: _______________________

Q3. Mamboly ve ianao? ENY* TSIA *Raha Eny, inona? ____________________________

1. _____________ 2. _____________ 3. _____________ 4. _____________ 5. _____________

Q4. Manana sarety ve? ENY TSIA Firy? _______________________________________

Q5: Inona no fomba fahandroana sakafo fampiasa ao an-trano? __________________________________________________ Q6. Inona ny jiro fampiasa ao an-trano? _________________________________Q7. Afa-po ve ianao amin’io karazanjiro io?

TENA FALY FALY TSISY AMBARA FALIFALY TSY FALY

Q8. Aiza no maka rano hosotroina/ ahandrahoana? ____________________________________________________________

Q9. Kojakoja ananana HERINARATRA [*Ataovy anaty boribory*] TV RADIO FRIGO

FIARA MOTO/SCOOTER BISIKILETA FAMANTARANANDRO FINDAY

Q10. Efitrano firy no misy ao an-tokantrano? _____________ Q11. Olona firy isan’efitra no miara-matory? _____________

Tafon-trano [*jereo*] RAFIA FANITSO BOZAKA SATRANA HAFA: ___________

Rindrina [*jereo*] TANY BARARATA HAZO BIRIKY SIMENITRA

Gorodona [*jereo*] TANY TREE-BARK HAZO SIMENITRA CARREAUX

**Fizarana 2: Firandraisan’ny zavaboahary sy ny biby fiompy**

Q1. Inona avy ny karazam- biby fiompy anananao ary firy? [*raha tsia dia dingano hatrany amin’ny Q16*]

|  | Isany | Arovanao ve izy ireo? ENY/TSIA | Fomba ahoana? |
| --- | --- | --- | --- |
| 1-Akoho  2- Zanak’akoho |  |  |  |
| Gana/Dokotra |  |  |  |
| Vorontsiloza |  |  |  |
| Osy /bengy |  |  |  |
| Kisoa /Lambo |  |  |  |
| Omby |  |  |  |
| Hafa |  |  |  |
|  |  |  |  |

Q2. Avelanao handehandeha FOANA ve ny akoho amam-borona? ENY TSIA

*[Raha Eny dia dingano hatrany amin’ny Q11. Raha tsia dia tohizo ny fanontaniana manaraka Mariho daholo raha mametraka ny akoho amam-borona amin’ny fotoana /toerana samihafa izy.]*

Q3. Inona no avy no tsy alefa mandehandeha?

AKOHO GANA/DOKOTRA VORONTSILOZA HAFA: ____________

Q4. Aiza no apetrakao ny akoho amam-borona? FISOKO AO AN-TRANO HAFA: _____________

Q4a. Hamafin’ny fisoko (1 = TSY MAFY, 5 = TENA MAFY)? ________ Sary # : ______________

Q5. Amin’ny fotoana manao ahoana no hidiana ny akoho amam-borona?

MARAINA ATOANDRO HARIVA ALINA

Q6. Amin’ny vaninandro inona no tsy avela hivoaka ny akoho amam-borona?

MAINTANY FAHAVARATRA IZY ROA

Q7. Maninona no hidianao ny akoho amam-borona? ___________________________________________________________

Q8. Mametraka fandrika hiarovana ny akoho amam-borona ve ianao?

ENY TSIA [*Raha tsia , dingano hatramin’ny Q11*]

Q8a. Aiza no asianao fandrika? ____________________________________________________________________________

Q9. Efa nahatratra biby tamin’ny fandrika ve ianao? ENY TSIA [*Raha eny dia fenoy ny tabilao eo ambany*]

| Inona ny biby efa tratranao/azonao? | Impiry teo amin’ny fiainanao ? | Impiry tanaty ny herintaona? | Tamin’ny fotoana? (maintany/orana/izy roa) | ** Tamin’ny fotoana inona? | Nataonao inona ilay biby? |
| --- | --- | --- | --- | --- | --- |
| Kary |  |  |  |  |  |
| Fosa |  |  |  |  |  |
| Jaboady |  |  |  |  |  |
|  |  |  |  |  |  |

** (Maraina,/Antoandro/Tolakanadro/Hariva)

Q10. Efa nisy olona teo an-tanana ve nahita biby tratra tao anaty fandrika natao hiarovana akoho amam-borona? ENY TSIA [*Raha eny dia fenoy ny tabilao eo ambany*]

| Iza/taiza? [*toerana/fotoana*] | Hitanao ve? | Biby inona | Impiry teo amin’ny fiainanao? | Impiry tao anaty ny herintaona? | ** Amin’ny fotoana inona? | Amin’ny vaninandro inona? (maintany/avy orana /izy roa) | Nataon’izy ireo inona ilay biby rehefa maty? |
| --- | --- | --- | --- | --- | --- | --- | --- |
|  |  |  |  |  |  |  |  |
|  |  |  |  |  |  |  |  |
|  |  |  |  |  |  |  |  |
|  |  |  |  |  |  |  |  |

[***Maraina/Atoandro.Hariva/Alina*]

Q11. Mahalala olona izay nanana akoho amam-borona efa novonoin’ny fosa ve ianao? ENY TSIA *[Raha eny dia tohizo, raha tsia dia dingano hatrany amin’ny Q17]*

| Iza/Taiza? [*toerana/fotoana*] | Hitanao ve? | Karazam-borona inona? | Ohatrinona no tokony ho vidiny? | Impiry teo amin’ny fiananao no nisehoan’izany? | Impiry no niseho tanaty ny herintaoan? | Amin’ny vaninandro inona? | Amin’ny fotoana inona ? | Nataon’izy ireo inona ilay biby rehefa maty? | Inona no nataon’izy ireo mba tsy hisehoan’izany intsony amin’ny ho avy? |
| --- | --- | --- | --- | --- | --- | --- | --- | --- | --- |
|  |  |  |  |  |  |  |  |  |  |
|  |  |  |  |  |  |  |  |  |  |
|  |  |  |  |  |  |  |  |  |  |
|  |  |  |  |  |  |  |  |  |  |

Q12. Mahalala olona hafa efa nihaza fosa ve ianao satria namomo ny akoho amam-boronao izy na noho ny antony hafa? ENY TSIA [Raha tsia, dingano hatramin’ny Q15]

Q12a. Hitanao ve? ENY TSIA (Raha tsia, dingano hatramin’ny Q14) Q12b: Oviana no nitranga? Taona: _____ _____ _____ Vaninandro: _____ _____ _____

Q12c: Raha Eny, nahoana? __________________________________________________________________________________Q12d. Impiry? _________________ ID: __

Q12e: Ahoana no nihazany fosa? ________________________________________________________________________________________________________________

Q13. Nahavoa ve izy nihaza fosa? ENY TSIA [Raha tsia, dingano hatramin’ny Q15] Q13a. Raha eny, fosa firy no voavono? ______________

Q13b. Taiza? __________________________________ Q13c.Natao inona ny fosa maty avy eo? _____________________________________________________________

Q14. Efa nanana akoho amam-borona maty/ voavono na tsy hita izay nalehany ve ianao? ENY TSIA Firy tamin’ny taon-dasa? _________________

Firy no nanjavona tsy hita, firy no hita fa maty? *[Raha tsia, dingano hatrany amin’ny Q16]*[Anontanio raha ireto biby manaraka ireto no namono ny akoho amam-borona, Inona raha tsy amin’ireo, *anontanio ny mombamomba ny tsirairay*]

| Inona no namono ny akoho amam-borona? | Biby inona no maty? | Ohatrinona no tokony ho vidiny? | Firy ny akoho amam-borona maty teo amin’ny fiainanao? | Firy no maty tanaty ny herintaona? | * Tamin’ny fotoana inona? | ** Amin’ny fotoana inona no tena isehoany? | Hitanao ve ny nisehoany? | Raha eny inona no nataonao? | Inona no nataonao mba tsy isehoan’iznay intsony amin’ny ho avy? |
| --- | --- | --- | --- | --- | --- | --- | --- | --- | --- |
| Fiara |  |  |  |  |  |  |  |  |  |
| Voromahery |  |  |  |  |  |  |  |  |  |
| Fosa |  |  |  |  |  |  |  |  |  |
| Aretina |  |  |  |  |  |  |  |  |  |
| Bibilava |  |  |  |  |  |  |  |  |  |
| Kary |  |  |  |  |  |  |  |  |  |
|  |  |  |  |  |  |  |  |  |  |
|  |  |  |  |  |  |  |  |  |  |
|  |  |  |  |  |  |  |  |  |  |

* *Avy orana/Maintany/Izy roa ** Maraina/Antoandro/Tolakandro/Hariva* [*Raha tsia dia dingano hatrany amin’ny Q17*]

Q15. Efa nihaza fosa ve ianaoa na ny olona ao an-tranonao satria namono ny akoho amam-boronao izy na noho ny antony hafa? ENY TSIA

Q15a. Oviana? Taona: ____ ____ ____ Vaninandro: ____ ____ ____ Q15b. Raha eny, nahoana? ______________________________________________________________

Q15c. Impiry? ______________ Q15d: Ahoana no nihazanao ny fosa? _________________________________________________________________________________

Q16. Nahavoa ve ianao na ny olona ao an-tokantranonao? ENY TSIA [*Raha tsia , dingano hatrany amin’ny Q17*

Q16a. Raha eny, firy ny fosa efa novonoinao? _____________ Q16b. Taiza no nitranga? _____________________________

Q16c. Nataonao inona ilay fosa rehefa maty? _________________________________________________________________

Q17. Manao ahoana ny fahitanao ny fosa?

TENA TIANAO TIANAO TSISY AMBARA TSY TIANAO TENA TSY TIANO

Q18. Inona no anton’izany? ______________________________________________________________________________

_____________________________________________________________________________________________________

Q19. Aminao, misy tombontsoa azo avy amin' ny fisian’ny fosa ve? ENY TSIA Inona ny tombony/ Nahoana no tsy misy tombony? ___________________________________________________________________________

Q20. Aminao, misy tombony ho an’ny forest tontolo iainana ve ny fosa? ENY TSIA Inona ny tombony/ Nahoana no tsy misy tombony?________________________________________________________________________________________

Q21. Aminao, inona ny antony mety mahatonga ny fosa hininana ny akoho amam-boronao?___________________________

_____________________________________________________________________________________________________

Q22. Matahotra fosa ve ianao sao dia mandratra mamono anao izy? ENY TSIA Nahoana?_________________

_____________________________________________________________________________________________________

Q23. Matahotra fosa ve ianao sao dia mihinana ny akoho amam-borona izy? ENY TSIA Nahoana?_____________

_____________________________________________________________________________________________________

Q24. Aminao, tokony ho ferana ve ny isan’ny fosa anaty ala? ENY TSIA Nahoana?_______________________

_____________________________________________________________________________________________________

Q25. Mino ny fady mifandray amin’ny fosa ve eto amin’ity tanàna ity? ENY TSIA

Raha eny dia inona avy? _________________________________________________________________________________

**Fizarana 3: Fihinanana hena**

Q1. Inona no tena sakafo haninareo matetika ao an-trano? ( afaka alahatrao ve?)

1. _____________ 2. _____________ 3. _____________ 4. _____________ 5. _____________

Q2. Misy fotoana ve ny fihinananareo azy ireo sa mandava-taona (Mba tanisao azafady) Maintany:_____________________________________________________________________________________________

Fahavaratra:___________________________________________________________________________________________
Izy roa:_______________________________________________________________________________________________

Q3. Inona no laoka tena fihinanareo ao an-trano? (Alaharo azafady)

1. _____________ 2. _____________ 3. _____________ 4. _____________ 5. _____________

Q4. Inona no voankazo tena fihinanareo ao an-trano? (Alaharo azafady)

1. _____________ 2. _____________ 3. _____________ 4. _____________ 5. _____________

Q5. Inona no hena tena fihinanareo? AVY AMIN’NY BIBY FIOMPY BIBY DIA (TSY OMPIANA)

Q6.Impiry isan-kerinandro ny sakafonareo no misy hena? Biby fiompy: ______ biby dia: ______ Tsy misy: ______

Q7. Isantaona, misy fotoana ahatsapanao tsy fahampiana ara-tsakafo ve ato an-tokantrano? ENY TSIA Fotoana inona? ___________Volana inona? ___________hatramin’ny___________

Q8. Inona ny hena avy amin’ny biby fiompy tena tianao? _______________________ Nahoana? _______________________

_____________________________________________________________________________________________________

***Aseho ny sary***

Q8a. Iza amin’ireo biby ireo no efa hitanao tao anaty ala? _______________________________________________________

_____________________________________________________________________________________________________

Q8b: Alaharo araka ny itiavanao azy ny hena efa nohaninao?

OMBY: ____ GANA/DOKOTRA: ____ OSY/BENGY: ____ AKOHO: ____

VORONTSILOZA/KOLOKA: ____ KISOA/LAMBO: ____ ONDRY: ______ HAFA: ______

Q8c. Inona no anton’ny nandaharanao azy toy izao?____________________________________________________________

_____________________________________________________________________________________________________

Q8d. Please rank in order of most consumed (only ranking what you have eaten):

OMBY: ____ GANA/DOKOTRA: ____ OSY/ BENGY: ____ AKOHO: ____ VORONTSILOZA/ KOLOKA: ____

KISOA/ LAMBO: ____ ONDRY: _____ HAFA: ____

Q8d. Inona no mahatonga anao hihinana hena avy amin’ny biby fiompy? __________________________________________

_____________________________________________________________________________________________________

­­­­­Q9. Efa nihinana ‘biby dia’ araka tahaka ireto amin’ny sary ireto ve ianao teo amin’ny fiainanao? ENY TSIA

[*Raha eny, valio ny Q9a, raha tsia , dingano hatrany amin’ny Q12*]

Q9a. Inona no biby dia tena tianao? ________________________ Nahoana? _______________________________________

_____________________________________________________________________________________________________

Q9b. Mba alaharo araka ny fitiavanao azy (ireo izay efa nohaninao ihany): Varika: ___ Vorona: ___

Trandraka: ___ Bibilava: ___ Lambo: ___ Biby mpihinan-kena (fosa, sns.): ___ Hazandrano: ___ Ramanavy: ___

Q9c. Inona no antony nandaharanao toy izao? ________________________________________________________________

_____________________________________________________________________________________________________

Q9d. Inona no mahatonga anao hihinana hena avy amin’ny biby dia? ______________________________________________

Q10. Mba alaharo araka ny fitiavanao azy (ireo izay efa nohaninao ihany) Varika/Gidro: ___ Vorona: ___

Trandraka: ___ Bibilava: ___ Lambo: ___ Biby mphinan-kena hafa (Foasa, sns): ___ Hazandrano: ___ Ramanavy: ___

Omby: ____ Gana/Dokotra: ____ Osy: ____ Akoho ____ Vorontsiloza: ____ Kisoa: ____

Q11. Inona avy ny biby efa nohaninao tamin’ny fiainanao?

| Biby | Karazam-biby | Impiry teo amin’ny fiainanao? | Impiry tao anatiny’ny taona lasa teo? | Impiry tao anatin’ny 7 andro farany teo? | Vaninandro inona no tena fihinananao hena (Maintany/Orana/Izy roa) | Ahoana no ahazoana ny hena? (Mividy/Mihaza/Fanomezana) | Aiza no mividy?/no mihaza?/ Iza no manome? | Raha mividy, ohatrinona /kg na / biby? | Nahoana no mividy (mora, matsiro,..?  Nahoana no mihaza? (Mora hita, tadiavina..?  Miny manonome (misy antony manokana? | Ahoana ny fomba fihazana? |
| --- | --- | --- | --- | --- | --- | --- | --- | --- | --- | --- |
|  |  |  |  |  |  |  |  |  |  |  |
|  |  |  |  |  |  |  |  |  |  |  |
|  |  |  |  |  |  |  |  |  |  |  |
|  |  |  |  |  |  |  |  |  |  |  |
|  |  |  |  |  |  |  |  |  |  |  |
|  |  |  |  |  |  |  |  |  |  |  |
|  |  |  |  |  |  |  |  |  |  |  |
|  |  |  |  |  |  |  |  |  |  |  |

Q12. Efa nihaza sy nivarotra ve ianao?

| Biby | Karazam-biby | Impiry teo amin’ny fiainanao? | Impiry tamin’ny taona lasa? | Vaninandro inona no tena famarotanao? | Ahoana no fomba hihazanao? | Impiry isan-kerinandro eo ianao no mihaza? | Amin’ny ankapobeny, biby firy no azonao isaky ny mandeha mihaza? | Aiza ianao no mihaza? | Aiza no hivarotanao azy avy eo? | Ohatrinona no hamarotanao azy /kg na /biby? | Inona no antony hohazanao sy hamarotanao? |
| --- | --- | --- | --- | --- | --- | --- | --- | --- | --- | --- | --- |
|  |  |  |  |  |  |  |  |  |  |  |  |
|  |  |  |  |  |  |  |  |  |  |  |  |
|  |  |  |  |  |  |  |  |  |  |  |  |

Q13. Inona no biby dia efa nohazainao be indrindra eo amin’ny fiainanao?

___________________________________________________________________________________

Q13a. Alaharo araka ny habetsahan’ny azonao (ny biby izay efa nohazainao ihany).

Varika: ___ Vorona: ___ Trandraka: ___ Bibilava: ___ Lambo: ___ Fosa: ___ Hazandrano: ___ Ramanavy: ___

Q13b. Inona no biby mora hazaina indrindra? ___________________________ Nahoana? __________

___________________________________________________________________________________

Q14. Misy fady mifandray amin’ny biby aty amin’ity toerana ity ve? ENY TSIA Fady inona? ________________________________________________________________________

**Fizarana 4. Fiarovana ny zavaboahary**

Q1. Ahoana ny hevitrao momba ny fiarovana ny zavaboahary? ________________________________

___________________________________________________________________________________

Q2. Nahoana ny olona no miaro ny zavaboahary? __________________________________________

___________________________________________________________________________________

Q3. Ho an’iza no iarovan’olona ny zavaboahary? Ahoana no mahatonga anao hieritreritra izany? ____

___________________________________________________________________________________

Q4. Tianao ve ny fiarovana ny zavaboahary?

TENA TIANAO TIANAO TSISY AMBARA TSY TIANAO TENA TSY TIANO

Q4a. Inona no anton’izany? ____________________________________________________________

___________________________________________________________________________________

Q4b. Mahazo tombony amin’ny fiarovana ny zavaboahary ve ianao? ENY TSIA

Inona? _____________________________________________________________________________

Q5. Fantatrao ve inona no atao hoe faritra voaaro ? ENY TSIA

Q5 Inona no dikan’izany? _____________________________________________________________

___________________________________________________________________________________

Q6 Inona araka ny hevitrao no antony niarovana na ny Ala? __________________________________

___________________________________________________________________________________

Q7Ahoana ny eritreritrao amin’ny fiarovana ity ala ho Parka Nasionaly ?

TENA TIANAO TIANAO TSY MAMPANINONA TSY TIANAO TENA TSY TIANO

Q7a. Inona no anton’izany? ____________________________________________________________

___________________________________________________________________________________

Q7b. Mahazo tombony amin’ity ala ity ve ianao? ENY TSIA Inona?________________

___________________________________________________________________________________

Q8 Efa nisy niresaka taminao momba ny fiarovana ny tontolo iainana ve? ENY TSIA

Iza? ________________________ Miasa amin’iza/inona izy ireo? ____________________________

Avy aiza izy ireo? ___________________________________________________________________

Inona no noteneniny anareo? ___________________________________________________________

Nanova ny fihevitrao momba ny fiarovana ny zavaboahary ve izany? ENY TSIA Nahoana? __________________________________________________________________________
