# Supplementary material for: Retaliatory killing and human perceptions of Madagascar’s largest carnivore and livestock predator, the fosa (Cryptoprocta ferox)
Source: PLoS One. 2019 Mar 15;14(3):e0213341. doi: 10.1371/journal.pone.0213341 (PMC6420034; doi:10.1371/journal.pone.0213341)
Supplement: S3 Table — The model variables included: Dependent Variable 1/2/3 (DV1, DV2, DV3); independent variable model 1/2/3 (IV1, IV2, IV3); Fixed Blocking Factor model 1/2/3 (BF1, BF2, BF3), and random effect model 1/2/3 (RE1, RE2, RE3). (DOCX) [file pone.0213341.s004.docx]

|  |  |  |  |
| --- | --- | --- | --- |
| **Variable** | **Description** | **Levels** | **Model** |
| Last Year Predation | Fosa predation of poultry previous year | Yes/No | DV1 |
| Fosa Attitude | Interviewee's attitude towards fosa | Strongly Dislike, Dislike, Neutral, Like, Strongly Like | DV2, IV3 |
| Retaliation | Retaliatory killing of fosa during interviewee's lifetime | Yes/No | DV3 |
| Village | The village where the household is located | Categorical Village Name | RE1, RE2, RE3 |
| Region | The geographical area the household is located | Boeny, Menabe, Moramanga, Vatovavy-Fitovinany, | BF1, BF2, BF3 |
| Forest Size | Size of the nearest primary forest (km^2^) | 1 - ∞ | IV1 |
| Village Size | Total number of households within the household's village | 1 - ∞ | IV1 |
| River Barrier | Presence of river between village and nearest forest | Yes/No | IV1 |
| Household Distance | Household distance to forest edge (m) | 1 - ∞ | IV1 |
| Flock Size | Total number of poultry owned by interviewee | 1 - ∞ | IV1 |
| Flock Coop | Coop used to protect poultry | Yes/No | IV1 |
| Snare | Snare used surrounding coop | Yes/No | IV1 |
| Poverty | Household wealth metric | 0 - 1 | IV2, IV3 |
| Education | Interviewee's highest level of education | None, Primary, Junior secondary, Senior secondary, Tertiary | IV2 |
| Poultry Owned | Poultry owned by household | 1 - ∞ | IV2 |
| Lifetime Predation | Fosa predation of poultry life time | Yes/No | IV2 |
| Conservation Experience | Has the interviewee been educated on the benefits of localised conservation | Yes/No | IV2, IV3 |
| Conservation Attitude | Interviewee's attitude towards conservation | Strongly Dislike, Dislike, Neutral, Like, Strongly Like | IV2, IV3 |
| Conservation Benefit | Does the interviewee receive any personal benefit from conservation | Yes/No | IV3 |
|  |  |  |  |
