# Supplementary material for: Retaliatory killing and human perceptions of Madagascar’s largest carnivore and livestock predator, the fosa (Cryptoprocta ferox)
Source: PLoS One. 2019 Mar 15;14(3):e0213341. doi: 10.1371/journal.pone.0213341 (PMC6420034; doi:10.1371/journal.pone.0213341)
Supplement: S4 Table — a) The total number of households that own poultry in deciduous and rainforests to have experienced fosa depredation (percentage of households in brackets) (Chi-squared test of independence, X2 = 121.67, df = 1, p < 0.01). b) The total number of households that own poultry to have experienced fosa depredation that house their poultry in a coop and not in a coop (percentage of households in brackets) (Chi-sq X2 = 0.69786, df = 1, p = 0.4035). (DOCX) [file pone.0213341.s005.docx]

a)

|  | **Predation** | **No Predation** |
| --- | --- | --- |
| **Deciduous** | 297 (42.1%) | 408 (57.9%) |
| **Rainforest** | 50 (11.3%) | 394 (88.7%) |

b)

|  | **Predation** | **No Predation** |
| --- | --- | --- |
| **Coop** | 299 (32.5%) | 620 (67.5%) |
| **No Coop** | 48 (36.6%) | 83 (63.4%) |
