# Supplementary material for: Retaliatory killing and human perceptions of Madagascar’s largest carnivore and livestock predator, the fosa (Cryptoprocta ferox)
Source: PLoS One. 2019 Mar 15;14(3):e0213341. doi: 10.1371/journal.pone.0213341 (PMC6420034; doi:10.1371/journal.pone.0213341)
Supplement: S5 Table — a) Model selection output for the highest weighed models of the predictors of a households’ likelihood to sustain fosa poultry predation. Preferred model is in bold. Degrees of freedom (df), log likelihood (logLik), Akaike’s Information Criterion (AICc), relative change in Akaike’s Information Criterion from top model (ΔAICc), and Akaike’s Information Criterion weight (AICcwt). b) The modelled output for the most parsimonious predictors, Snare and Region. P-value (Pr (>|z|)) at significance level (p < 0.001***, p < 0.01 **, p < 0.05 *). (DOCX) [file pone.0213341.s006.docx]

a)

|  |  |  |  |  |  |
| --- | --- | --- | --- | --- | --- |
| **Model** | **Df** | **logLik** | **AICc** | **Δ AICc** | **AIC_c_wt** |
| Region + Snare + Forest Size | 7 | -519.38 | 1052.85 | 0 | 0.15 |
| Region + Snare + Village Size | 7 | -519.75 | 1053.6 | 0.75 | 0.11 |
| **Region + Snare** | **6** | **-520.96** | **1053.99** | **1.14** | **0.09** |
| Region + River + Snare | 7 | -519.95 | 1054 | 1.15 | 0.09 |
| Region + Forest Size + Village Size | 7 | -520.24 | 1054.58 | 1.73 | 0.06 |
| Region + Forest Size | 6 | -521.54 | 1055.16 | 2.31 | 0.05 |
| Region + Village Size | 6 | -521.61 | 1055.29 | 2.44 | 0.05 |
|  |  |  |  |  |  |
| b) |  |  |  |  |  |
| **Variables** | **Estimate** | **Std. Error** | **z value** | **Pr (>\|z\|)** |  |
| (Intercept) | -1.3786 | 0.3172 | -4.347 | 1.38e-05 *** |  |
| Menabe Region | 0.4549 | 0.4535 | 1.003 | 0.3158 |  |
| Moramanga Region | -1.2513 | 0.4762 | -2.628 | 0.0086 ** |  |
| Vatovavy-Fitovinany Region | -1.958 | 0.6621 | -2.958 | 0.0031 ** |  |
| Snare (Yes) | 0.8333 | 0.3993 | 2.087 | 0.0369 * |  |
|  |  |  |  |  |  |
