# Supplementary material for: Retaliatory killing and human perceptions of Madagascar’s largest carnivore and livestock predator, the fosa (Cryptoprocta ferox)
Source: PLoS One. 2019 Mar 15;14(3):e0213341. doi: 10.1371/journal.pone.0213341 (PMC6420034; doi:10.1371/journal.pone.0213341)
Supplement: S6 Table — (DOCX) [file pone.0213341.s007.docx]

| **Question** | **Yes: Reason** | **Total Respondents** | **%** | **No: Reason** | **Total Respondents** | **%** |
| --- | --- | --- | --- | --- | --- | --- |
| Do fosa provide any benefit? | Rat predator | 100 | 23.9 | Poultry predator | 540 | 44.85 |
|  | Tourism revenue | 88 | 21.2 | No benefit | 369 | 30.65 |
|  | Ecosystem component | 70 | 15 | Don't know | 128 | 10.6 |
|  | Prevents human forest entry | 46 | 11 | Other | 167 | 13.9 |
|  | Other | 121 | 28.9 |  |  |  |
| Are you scared of fosa for your poultry? | Fosa are persistent | 210 | 28 | Village geographically protected | 212 | 33.1 |
|  | Fosa are carnivorous | 144 | 19.2 | Don't own poultry | 178 | 27.7 |
|  | Fosa scare poultry | 80 | 10.7 | Own dog | 61 | 9.5 |
|  | Chickens are easily killed | 67 | 8.9 | Coop is secure | 57 | 8.9 |
|  | Other | 249 | 33.2 | Other | 133 | 20.8 |
| Should fosas' population be controlled? | Overpopulation means greater depredation | 169 | 46.6 | God's creation | 309 | 31.3 |
|  | Fosa kill poultry | 69 | 19 | Control isn't feasible | 163 | 16.5 |
|  | Other | 125 | 34.4 | Fosa should be allowed to survive | 124 | 12.6 |
|  |  |  |  | Other | 390 | 39.6 |
