# Supplementary material for: Retaliatory killing and human perceptions of Madagascar’s largest carnivore and livestock predator, the fosa (Cryptoprocta ferox)
Source: PLoS One. 2019 Mar 15;14(3):e0213341. doi: 10.1371/journal.pone.0213341 (PMC6420034; doi:10.1371/journal.pone.0213341)
Supplement: S7 Table — a) Model selection output for the highest weighed models containing the predictors of a households’ attitude towards fosas. Preferred model is in bold. Degrees of freedom (df), log likelihood (logLik), Akaike’s Information Criterion (AICc), relative change in Akaike’s Information Criterion from top model (ΔAICc), and Akaike’s Information Criterion weight (AICcwt). b) The modelled output for the most parsimonious predictors, Education, Lifetime Predation and Region. P-value (Pr (>|z|)) at significance level (p < 0.001***, p < 0.01 **, p < 0.05 *). (DOCX) [file pone.0213341.s008.docx]

a)

| **Model** | **df** | **logLik** | **AICc** | ΔAICc | AIC_c_wt |
| --- | --- | --- | --- | --- | --- |
| Conservation Benefit + Education + Lifetime Predation + Region | 10 | -1346.39 | 2713.01 | 0 | 0.18 |
| Conservation Benefit + Education + Lifetime Predation + Poultry Owned + Region | 11 | -1345.82 | 2713.91 | 0.9 | 0.12 |
| **Education + Lifetime Predation + Region** | **9** | **-1347.93** | **2714.04** | **1.03** | **0.11** |
| Conservation Benefit + Education + Conservation Experience + Lifetime Predation + Region | 11 | -1346.23 | 2714.73 | 1.72 | 0.08 |
| Education + Lifetime Predation + Poultry Owned + Region | 10 | -1347.27 | 2714.77 | 1.76 | 0.08 |
| Conservation Benefit + Education + Lifetime Predation + Region + Poverty | 11 | -1346.29 | 2714.85 | 1.84 | 0.07 |
| Conservation Benefit + Conservation Attitude + Education + Lifetime Predation + Region | 11 | -1346.38 | 2715.03 | 2.01 | 0.07 |
| Education + Conservation Experience + Lifetime Predation + Region | 10 | -1347.67 | 2715.57 | 2.55 | 0.05 |
| Education + Lifetime Predation + Region + Poverty | 10 | -1347.83 | 2715.88 | 2.87 | 0.04 |
| Conservation Attitude + Education + Lifetime Predation + Region | 10 | -1347.9 | 2716.02 | 3.01 | 0.04 |
| Education + Conservation Experience + Lifetime Predation + Poultry Owned + Region | 11 | -1346.95 | 2716.18 | 3.16 | 0.04 |
| Education + Lifetime Predation + Poultry Owned + Region + Poverty | 11 | -1347.12 | 2716.51 | 3.5 | 0.03 |
| Conservation Attitude + Education + Lifetime Predation + Poultry Owned + Region | 11 | -1347.26 | 2716.78 | 3.77 | 0.03 |
| Education + Conservation Experience + Lifetime Predation + Region + Poverty | 11 | -1347.57 | 2717.41 | 4.4 | 0.02 |
| Conservation Attitude + Education + Conservation Experience + Lifetime Predation + Region | 11 | -1347.64 | 2717.54 | 4.53 | 0.02 |
| Conservation Attitude + Education + Lifetime Predation + Region + Poverty | 11 | -1347.77 | 2717.8 | 4.79 | 0.02 |

b)

| **Variables** | **Estimate** | **Std. Error** | **z value** | **Pr(>\|z\|)** |
| --- | --- | --- | --- | --- |
| Lifetime Predation | -0.90432 | 0.15114 | -5.983 | p < 0.001 *** |
| Moramanga Region | -0.92748 | 0.17582 | -5.275 | p < 0.001 *** |
| Vatovavy-Fitovinany Region | -0.38755 | 0.18304 | -2.117 | 0.034237 * |
| Education | 0.25712 | 0.07191 | 3.576 | 0.000349 *** |
