# Supplementary material for: Retaliatory killing and human perceptions of Madagascar’s largest carnivore and livestock predator, the fosa (Cryptoprocta ferox)
Source: PLoS One. 2019 Mar 15;14(3):e0213341. doi: 10.1371/journal.pone.0213341 (PMC6420034; doi:10.1371/journal.pone.0213341)
Supplement: S8 Table — a) Model selection output for the highest weighed models containing the predictors of a households’ retaliatory killing of a fosa. Preferred model is in bold. Degrees of freedom (df), log likelihood (logLik), Akaike’s Information Criterion (AICc), relative change in Akaike’s Information Criterion from top model (ΔAICc), and Akaike’s Information Criterion weight (AICcwt). b) The modelled output for the most parsimonious predictors, Fosa Attitude, Poverty and Region. P-value (Pr (>|z|)) at significance level (p < 0.001***, p < 0.01 **, p < 0.05 *). (DOCX) [file pone.0213341.s009.docx]

| a) |  |  |  |  |  |
| --- | --- | --- | --- | --- | --- |
| **Model** | **df** | **logLik** | **AICc** | **Δ** | **Weight** |
| **Fosa Attitude + Region + Poverty** | **6** | **-85.02** | **182.12** | **0** | **0.43** |
| Fosa Attitude + Conservation Benefit + Region + Poverty | 7 | -84.69 | 183.48 | 1.36 | 0.22 |
| Fosa Attitude + Conservation Education + Region + Poverty | 7 | -84.93 | 183.96 | 1.84 | 0.17 |
| Fosa Attitude + Conservation Attitude + Region + Poverty | 7 | -84.98 | 184.05 | 1.93 | 0.16 |
| Region + Poverty | 5 | -90.28 | 190.61 | 8.49 | 0.01 |
|  |  |  |  |  |  |
| b) |  |  |  |  |  |
| **Variables** | **Estimate** | **Std. Error** | **z value** | **Pr(>\|z\|)** |  |
| (Intercept) | -1.9295 | 0.8198 | -2.354 | 0.018593 * |  |
| Moramanga Region | -1.2416 | 0.8765 | -1.417 | 0.156593 |  |
| Vatovavy-Fitovinany Region | -2.7201 | 1.3198 | -2.061 | 0.039299 * |  |
| Fosa Attitude | -0.9413 | 0.3244 | -2.902 | 0.003706 ** |  |
| Poverty | -1.1361 | 0.3397 | -3.345 | 0.000824 *** |  |
